# Night eating model shows time-specific depression-like behavior in the forced swimming test

**DOI:** 10.1038/s41598-018-19433-8

**Published:** 2018-01-18

**Authors:** Atsushi Haraguchi, Miyabi Fukuzawa, Shiho Iwami, Yutaro Nishimura, Hiroaki Motohashi, Yu Tahara, Shigenobu Shibata

**Affiliations:** Laboratory of Physiology and Pharmacology, School of Advanced Science and Engineering, Waseda University, Tokyo, Japan

## Abstract

The circadian clock system is associated with feeding and mood. Patients with night eating syndrome (NES) delay their eating rhythm and their mood declines during the evening and night, manifesting as time-specific depression. Therefore, we hypothesized that the NES feeding pattern might cause time-specific depression. We established new NES model by restricted feeding with high-fat diet during the inactive period under normal-fat diet *ad libitum*. The FST (forced swimming test) immobility time in the NES model group was prolonged only after lights-on, corresponding to evening and early night for humans. We examined the effect of the NES feeding pattern on peripheral clocks using PER2::LUCIFERASE knock-in mice and an *in vivo* monitoring system. Caloric intake during the inactive period would shift the peripheral clock, and might be an important factor in causing the time-specific depression-like behavior. In the NES model group, synthesis of serotonin and norepinephrine were increased, but utilization and metabolism of these monoamines were decreased under stress. Desipramine shortened some mice’s FST immobility time in the NES model group. The present study suggests that the NES feeding pattern causes phase shift of peripheral clocks and malfunction of the monoamine system, which may contribute to the development of time-specific depression.

## Introduction

The mammalian circadian clock system has an approximately 24 h rhythm and regulates various physiological functions, including metabolism, feeding cycle, and sleep-wake cycle^1^. The mammalian circadian clock system is assembled from clock genes, including *Cry1/2*, *Per1/2*, *Bmal1*, and *Clock*, which generate an approximately 24 h rhythm by building a transcriptional and translational feedback loop^2^. Mammals possess a central clock and peripheral clocks^3^. The light-dark (LD) cycle entrains the central clock, which is located in the suprachiasmatic nucleus (SCN) in the hypothalamus^4^. Subsequently, the central clock orchestrates the peripheral clocks, which are located in all organs^5^. Peripheral clocks also are entrained by other stimulations, such as exercise, physical and psychological stress, and feeding^6–8^. Regarding feeding in particular, the timing and composition of food are important regulators for peripheral clocks^9–11^, whereas the central clock regulates the feeding rhythm by controlling the secretion rhythm of feeding related hormones, including leptin, orexin, and ghrelin^12–14^. Taken together, these studies indicate a close interaction between the circadian clock system and feeding rhythm.

The circadian clock system of patients with eating rhythm disorders, such as night eating syndrome (NES) and sleep-related eating disorder (SRED), has not been investigated, although we have determined that the circadian clock system would interact with the feeding rhythm. Patients with NES and SRED have episodes of night eating with and without consciousness, respectively^15^. NES is diagnosed by various criteria^16^ and is listed in the *Diagnostic and Statistical Manual of Mental Disorders* 5^th^ edition. SRED was conceptualized in 1991^17^. One of the characteristics of patients with NES is that their mood declines during the evening and night, contrary to the usual pattern found in depression^18^. On the other hand, patients with SRED exhibit more symptoms of depression^19^. Accordingly, these studies indicate that night eating, which is referred to as disturbed feeding rhythm, may be associated with time-specific and nonspecific depression.

**Figure 1:**
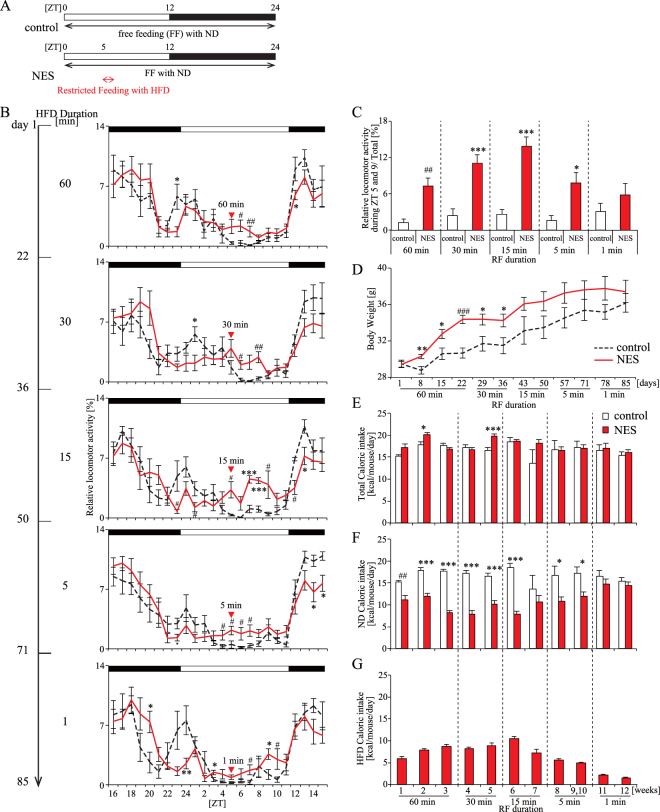
Effect of duration of RF with HFD at ZT 5 on locomotor activity, body weight, and caloric intake (Experiment 1). (**A**) Feeding schedules of the control and NES model groups. Mice in the control group were fed with ND throughout the day. Mice in the NES model groups were fed with ND throughout the day and restricted feeding with HFD at ZT 5. (**B**) Relative locomotor activity rhythm at each time point of RF with HFD at ZT 5 (60, 30, 15, 5, and 1 min). Red triangles represent the start of RF. Open and closed bars indicate light and dark periods, respectively. (**C**) Sum of relative locomotor activity during ZT 5 and 9. (**D**) Increase in body weight. (**E**–**G**) Change in average caloric intake of total (**E**), ND (**F)**, and HFD (**G**) at each RF duration. Data are presented as the mean ± standard error of the mean (SEM; control group, *n* = 6; NES model group, *n* = 8). *p < 0.05, **p < 0.01, ***p < 0.001 vs. the control group (Student’s *t*-test); ^#^*p* < 0.05, ^##^*p* < 0.01, ^###^*p* < 0.001 vs. the control group (Mann-Whitney test).

In the present study, we hypothesized that the NES feeding pattern might contribute to the development of time-specific depression through the disturbance of the circadian clock system. We were interested in evening depression in patients with NES and could not easily establish a model of night eating without consciousness, as shown in SRED. Many previous studies have prepared mutant or knock-out mice, and restricted feeding schedule (RF) during the inactive period as models of changed/delayed feeding rhythm^20–23^. If we used these mice and this RF schedule, we could not clearly distinguish the effect of disturbed feeding rhythm on the time-specific depression-like behavior from the effect of mutation, knock-out, and RF themselves on the depression-like behavior. Gene mutation, knock-out, and acute forced fasting affect the depression-like behavior^24,25^. Therefore, we needed to establish a new NES model mouse using wild-type mice without a fasting period. At first we established a new NES mouse model fed with high-fat diet (HFD) for a short duration over the inactive period under *ad libitum* feeding with normal-fat diet (ND) (Fig. 1A). In our previous study, we described that mice ate HFD at Zeitgeber time (ZT) 5 (lights-on time was defined as ZT 0) under a 2-h RF with HFD from ZT 5, although mice ate ND during the active period^26^. This feeding pattern was done to simulate human NES, which is characterized by a delayed phase of the circadian feeding pattern^27^.

Depression may be caused by dysfunction in the monoamine systems of serotonin (5-hydroxytryptamine, 5-HT) and norepinephrine (NE), as previous studies have shown that selective serotonin reuptake inhibitors (SSRIs), serotonin norepinephrine reuptake inhibitors, and other monoamine reuptake inhibitors act as antidepressants^28–30^. Studies in mice showed that the 5-HT metabolic rate (5-HIAA/5-HT, 5-HIAA is the 5-HT metabolite) is increased in mice showing depression-like behavior^31^, and the aforementioned antidepressants decrease the 5-HT metabolic rate in the hippocampus^32^. In addition, a previous study indicated the relationship between depression and a hypofunction of the noradrenergic system, and the effect of some antidepressants on the NE and the NE metabolite, MHPG^33^. Human studies showed that some drugs, such as topiramate, sertraline, and escitalopram, could improve depressive symptoms in approximately half of the patients with NES^34,35^. Moreover, a study in mice showed that long-term feeding with HFD causes depression-like behavior^36^, and a human study showed that balanced meals, including fruit, vegetables, and olive oil, reduce the risk of development of depression^37^. However, the effect of disturbed feeding rhythms, such as the eating pattern of patients with NES, on depression and monoamine systems has not been investigated.

In this study, our aims were to establish a new NES model without fasting and using wild-type mice, confirm its validity, examine the relationship between the disturbed feeding rhythm and the time-specific depression-like behavior, and finally to elucidate the cause of time-specific depression-like behavior from the perspectives of the circadian clock and monoamine systems. Thus, we conducted five main experiments. Firstly, we established the model of disturbed feeding rhythm similar to the eating pattern of patients with NES (NES model). Secondly, we evaluated the effect of disturbed feeding rhythm on time-specific depression using a forced swimming test (FST), which is an established method of measuring depression-like behavior^38^. Thirdly, we examined the NES feeding pattern causing the time-specific depression-like behavior from the perspective of the circadian clock system. Fourthly, we examined monoamine metabolism in the hippocampus and striatum before and after the FST. Lastly, we evaluated the effect of desipramine on the prolonged FST immobility time caused by the NES feeding pattern.

## Results

### Five-minute RF with HFD is an adequate feeding schedule to mimic the eating pattern of patients with NES (Experiment 1)

To establish a new NES model, female mice in the NES model group were kept under RF with HFD from ZT 5 under ND free feeding conditions (Fig. 1A). We decided upon this feeding schedule as the NES eating pattern, which is characterized by a delayed phase of the circadian eating pattern^27^. Our previous study showed that mice attained more than 30% of their total caloric intake intake from a 2-h RF with HFD^26^. To determine an adequate duration of RF with HFD, we explored the effect of RF duration on caloric intake and locomotor activity rhythm, and examined the feeding condition that resulted in HFD caloric intake that was approximately 25% of the total caloric intake, and which affected locomotor activity. Mice in the NES model group showed activity during the middle of the inactive period when RF was stopped after mice maintained on each feeding condition for more than 2 weeks, while the control group showed less activity. When the RF duration was 5–60 min, locomotor activity in the NES model group was increased compared with that in the control group during the middle of the inactive period (Fig. 1B,C). Body weight in the NES model group increased compared with that in the control group (Fig. 1D). In the NES model group, ND caloric intake was decreased compared with that in the control group, because mice in the NES model group consumed HFD during RF. Total caloric intake in the NES model group was increased compared to the control group when the RF duration was 60 and 30 min, and total caloric intake in both groups was similar when the RF duration was less than 15 min (Fig. 1E,F). In addition, mice in the NES model group consumed more than 25% of their total caloric intake during RF with HFD when the duration of RF was 5–60 min, and their HFD caloric intake was approximately 25% of their total caloric intake when the RF duration was 5 min (Fig. 1G). These results indicate that RF of 5 min was the best feeding schedule to establish a NES model.

To confirm whether the control feeding condition was optimal and whether the start timing of RF was important, we performed further experiments. First, to confirm whether the control feeding condition was optimal, we added two groups. The NES-ZT5-ND group had a 5-min RF with ND at ZT 5 and FF with ND. The NES-ZT17-HFD group had a 5-min RF with HFD at ZT 17 and FF with ND (Figure S1A). Total caloric intake in the four groups was similar and ND caloric intake in the NES-ZT17-HFD group was significantly lower than that in the other groups (Figure S1B). Body weight in the NES-ZT5-ND group was higher than that in the control and NES-ZT17-HFD groups, and lower than that in the NES-ZT5-HFD (NES model) group, although there were no significant differences in body weight in week 5 (Figure S1C). Locomotor activity in the control, NES-ZT5-ND, and NES-ZT17-HFD groups displayed a similar rhythm (Figure S1D).

Next, to confirm whether the start timing of RF was important, we added two groups. The NES-ZT1 group had a 5-min RF with HFD at ZT 1 and FF with ND. The NES-ZT9 group had a 5-min RF with HFD at ZT 9 and FF with ND (Figure S2A). Total caloric intake in the three groups was similar and ND caloric intake in the NES-ZT9 group was lower than that in the other groups, although there were no significant differences in caloric intake between the groups (Figure S2B). Body weight in the NES-ZT1 and NES-ZT9 groups was higher than that in the control group, although there were no significant differences in body weight (Figure S2C). Locomotor activity in the control and NES-ZT1 groups displayed a similar rhythm. Relative locomotor activity levels in the NES-ZT9 groups were increased at ZT 7–8 compared with those in the control group, although the values at other time points were similar to those in the control group (Figure S2D). These results indicate that FF with ND is one optimal control feeding condition, and that RF with HFD at ZT 5 is an adequate NES model feeding condition.

### Five-minute RF with HFD increases locomotor activity and caloric intake during the inactive period up to week 12 (Experiment 2)

**Figure 2:**
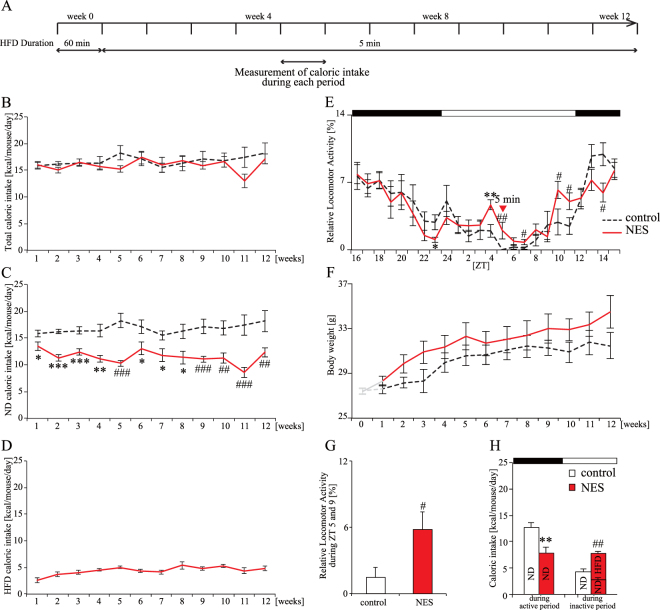
Effect of 5-min RF with HFD at ZT 5 on locomotor activity, body weight, and caloric intake (Experiment 2). (**A**) Experimental schedule. (**B**–**D**) Change in average caloric intake of total (**B**), ND (**C**), and HFD (**D**) under 5-min RF. (**E**) Relative locomotor activity rhythm under 5-min RF with HFD at ZT 5. Red triangle represents the start of RF. Open and closed bars indicate light and dark periods, respectively. (**F**) Increase in body weight. Gray period indicates under-habituation. (**G**) Sum of relative locomotor activity during ZT 5 and 9. (**H**) Caloric intake during each period for ND and HFD. ND and HFD are shown in every bar. Data are presented as the mean ± SEM (*n* = 8). *p < 0.05, **p < 0.01, ***p < 0.001 vs. the control group (Student’s *t*-test); ^#^*p* < 0.05, ^##^*p* < 0.01, ^###^p < 0.001 vs. the control group (Mann-Whitney test).

To analyze the long-term effect of a 5-min RF with HFD on the feeding pattern, body weight, and locomotor activity, the control and NES model groups of mice were fed for 3 months (Fig. 2A). Feeding in the NES model group featured the HFD for 1-h in the first week as a habituation period. Both groups had similar amounts of total caloric intake, and mice in the NES model group consistently consumed about 25% of their total caloric intake during RF with HFD from week 4 (Fig. 2B–D). Moreover, the NES model group had a lower caloric intake during the active period and a higher caloric intake during the inactive period than the control group at week 5 (Fig. 2H). Compared with the control group, the NES model group displayed increased locomotor activity during the middle of the inactive period (Fig. 2E,G). These results indicate that the feeding pattern and locomotor activity would be consistently observed in mice with a 5-min RF for 4 weeks and up to 12 weeks.

Body weight in the NES model group was increased compared to that in the control group, but there were no significant differences (Fig. 2F). We also monitored the daily respiratory exchange ratio (RER) rhythm and daily energy expenditure (EE) rhythm (Figure S3). The RER levels during the inactive period in the NES model group were higher than those in the control group (Figure S3A). There was no significant difference in RER levels between during the inactive and active periods in the NES model group, although there was a significant difference in RER levels in the control group (Figure S3C). The EE levels in the NES model group were higher than those in the control group (Figure S3B and S3D). Body weight in the NES model group was higher than that in the control group, although total caloric intake in both groups was similar and the EE levels in the NES model group was higher than in the control group. These results indicated that NES feeding pattern might cause body weight gain.

### Five-minute, but two-minute, RF with HFD affects the phase of peripheral clocks (Experiment 3)

**Figure 3:**
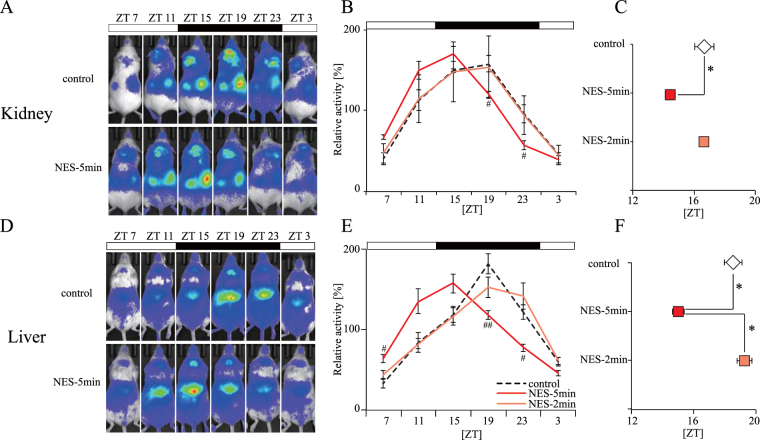
Effect of RF duration of 5-min and 2-min with HFD at ZT 5 on peripheral clocks (Experiment 3). (**A**–**F**) Representative images (**A** and **D**), analyzed wave forms (**B** and **E**), and peak phases (**C** and **F**) of *in vivo* PER2::LUC bioluminescence in the kidney (**A**–**C**) and the liver (**D**–**F**). Open and closed bars indicate light and dark periods, respectively. Data are presented as the mean ± SEM (control group, *n* = 6; NES-5 min group, *n* = 5; NES-2 min group, *n* = 3). *p < 0.05 (one-way ANOVA with Tukey’s multiple comparison test). ^#^*p* < 0.05, ^##^*p* < 0.01 vs. the control group (Kruskal-Wallis test with Dunn’s multiple comparison test).

To examine the effect on the peripheral clocks of RF with a duration of 5-min with HFD (NES-5 min), which was demonstrated in Experiment 1 to be an adequate feeding schedule for the NES model group, and RF with a duration of 2-min (NES-2min), which is a feeding schedule for consumption of a lower caloric intake from HFD, we monitored the bioluminescence rhythm in the kidney and liver using PER2::LUCIFERASE (PER2::LUC) knock-in mice^39,40^. We monitored the peripheral clocks in the kidney and the liver using an *in vivo* monitoring system after 2- and 5-min HFD feeding for 4 weeks. The kidney and the liver PER2 expression rhythms in the NES-5 min group, but not the NES-2 min group, were advanced compared with those in the control group (Fig. 3). We also monitored PER2::LUC bioluminescence rhythm in the SCN and liver *ex vivo*. Similar to the above results, the peak 1 phase was advanced in the liver in the NES-5min (NES model) group compared with that in the control group. However, the peak 1 phase was similar in the SCN in both groups (Figure S4). Taken together, the observations indicate that the amount of caloric intake during the inactive period with a 5-min RF stimulated the entrainment of the peripheral clocks only.

### Five-minute RF with HFD increases the FST immobility time only at ZT 1, the early time of the inactive period (Experiment 4)

**Figure 4:**
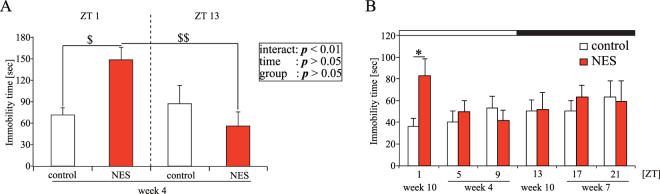
Effect of 5-min RF with HFD at ZT 5 on FST immobility time (Experiment 4). (**A**) The FST immobility time at ZT 1 and 13 in mice after 4 weeks of each feeding condition. The results of two-way ANOVA are presented on the right hand side of the graph. (**B**) The FST immobility time at ZT 1, 5, 9, 13, 17, and 21. The FST was conducted after 4 weeks of each feeding condition, with and interval between each FST of 2 weeks. The FST at ZT 5 and 9 were conducted in week 4, the FST at ZT 17 and 21 were conducted in week 7, and the FST at ZT 1 and 13 were conducted in week 10. Open and closed bars indicate light and dark periods, respectively. Data are presented as the mean ± SEM (the control group, *n* = 6; the NES model groups, *n* = 7). *p < 0.05 vs. the control group (Student’s *t*-test). ^$^*p* < 0.05, ^$$^*p* < 0.01 (two-way ANOVA with Tukey’s multiple comparison test).

To examine the effect of 5-min RF with HFD on depression-like behavior, we examined the immobility time using the FST in mice under the NES feeding pattern for several weeks. The experiment 2 demonstrated that the feeding pattern and locomotor activity in the NES model group were stable under the NES feeding pattern for 4 weeks. Thus, we first examined the FST immobility time at ZT 1 and 13 using different mice after each feeding condition for 4 weeks. The FST immobility time at ZT 1 in the NES model group was prolonged compared with that in the control group (Fig. 4A). Mice were placed in water for 15 min as a pre-test (pre-FST) 24 h before mice were placed in water for 6 min (FST). To examine whether habituation affected the FST immobility time, we examined the pre-FST immobility time. The change of immobility time in the pre-FST was similar to that in the FST, with no significant differences (Figure S5). Accordingly, in further experiments, we evaluated the immobility time in the FST only.

We monitored the FST immobility time at six different ZTs, with 2-week interval of FST. This protocol was to ensure that the FST immobility time was not influenced by the previous FST. We divided the control and NES model groups into two groups. In one group, we conducted the FST at ZT 5 in week 4, at ZT 17 in week 7, and ZT 1 in week 10. In the other group, we conducted the FST at ZT 9 in week 4, at ZT 21 in week 7, and ZT 13 in week 10. The FST immobility time in the NES model group was prolonged when examined at ZT 1 only, even after repeated FST treatment (Fig. 4B). To examine whether memory affected the FST immobility time, we prepared two additional sets of control and NES model groups for measurements of immobility time in the FST at ZT 9 and 17 after the NES feeding model for 4 weeks. The FST immobility times at ZT 9 and 17 in the NES model group were similar to those in the control group (Figure S6A and S6B). In other words, in this study, a 2-week interval was an adequate length of time to extinguish the effect of previous FST on immobility time. These results suggested that RF with 5-min duration with HFD increased the FST immobility time at ZT 1 regardless of the previous FST experience. Examination of the FST immobility time in the NES-ZT5-ND, NES-ZT17-HFD, NES-ZT1, and NES-ZT9 groups revealed a similar FST immobility time in these groups compared to that in the control group (Figure S1E and S2E).

We prepared another group of NES model mice to confirm these results. The effect of the 5-min RF on the FST immobility time was observed when mice were treated with this RF for a prolonged period (12 weeks) (Figure S6C). The 5-min RF with HFD only at ZT 5 prolonged the FST immobility time only at ZT 1, which was referred to as time-specific depression-like behavior.

Furthermore, we evaluated the depression-like behavior using another behavior test, namely the tail suspension test (TST)^41^. However, when we conducted the TST at ZT1, the TST immobility time in the NES model group was similar to that in the control group (Figure S7).

Lastly, we evaluated serum corticosterone and serum leptin, since corticosterone is a stress marker and leptin is involved in eating HFD^42,43^. Both hormone levels at ZT 1 and 13 in the NES model group were similar to those in the control group (Figure S8). In the NES model group, the ratio of serum corticosterone level at ZT 13 to that at ZT 1 was lower than that in the control group. Taken together, these results indicate that the 5-min RF with HFD only at ZT 5 prolonged the immobility time only in the FST at ZT 1, and decreased the ratio of serum corticosterone level at ZT 13 to ZT 1.

### Five-minute RF with HFD produces inadequate locomotor activity, affects caloric intake, and prolongs the FST immobility time (Experiment 5)

**Figure 5:**
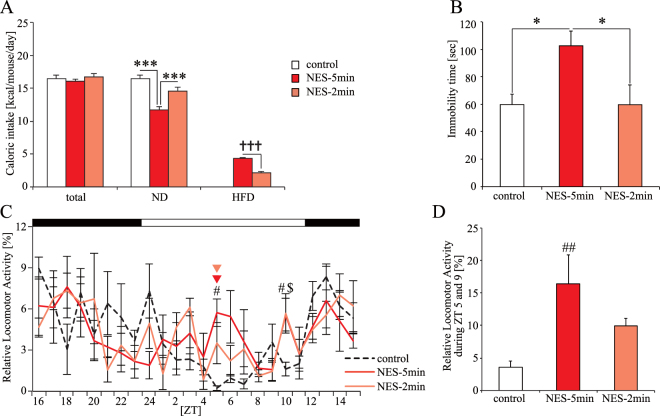
Effect of RF duration of 5-min and 2-min with HFD at ZT 5 on caloric intake, locomotor activity, and FST immobility time at ZT 1 (Experiment 5). (**A**) Average caloric intake of total, ND, and HFD under each feeding condition. (**B**) The FST immobility time at ZT 1. Data are presented as the mean ± SEM (control group, *n* = 6; NES-5 min and 2 min groups, *n* = 7). (**C**) Relative locomotor activity rhythm under each feeding condition. Red and orange triangles represent the commencement of RF. Open and closed bars indicate light and dark periods, respectively. (**D**) Sum of relative locomotor activity during ZT 5 and 9. Data are presented as the mean ± SEM (control group, *n* = 6; NES-5 min and 2 min groups, *n* = 4). *p < 0.05, ***p < 0.001 (one-way ANOVA with Tukey’s multiple comparison test); ^†††^*p* < 0.001 (Student’s *t*-test). ^#^*p* < 0.05, ^##^*p* < 0.01 the NES-5 min vs. the control group; ^$^*p* < 0.05 the NES-2 min vs. the control group (Kruskal-Wallis test with Dunn’s multiple comparison test).

To examine whether the 5-min RF with HFD was effective in causing the time-specific depression-like behavior, we examined a 2-min RF with HFD (NES-2 min) group in addition to the control and NES model (NES-5 min) groups. The ND caloric intake in the control and NES-2 min groups were similar. In contrast, the ND caloric intake and HFD caloric intake in the NES-5 min group were decreased and increased, respectively (Fig. 5A). Similar to previous data (Fig. 4), the FST immobility time at ZT 1 in the NES-5 min group was significantly prolonged compared with that in other groups (Fig. 5B). In addition, we monitored the locomotor activity of the mice (Fig. 5C), and found that locomotor activity during the middle of the inactive period in the NES-5 min group was increased compared with that in the control and NES-2 min groups, with a significant difference between the NES-5 min and the control groups (Fig. 5D). Furthermore, we sought to confirm whether the NES feeding pattern caused time-specific depression-like behavior in male mice. caloric intake intake, body weight, and locomotor activity rhythm in male mice were similar to those in female mice (Figure S9A–S9D). The FST immobility time at ZT 1 in the NES model group was significantly higher than that in the control group using male mice (Figure S9E). These results suggest that the NES feeding pattern can prolong the FST immobility time at ZT 1 regardless of gender. We observed that the peak phase of peripheral clocks in the NES-5 min group was significantly advanced compared to that in other groups *in vivo* and *ex vivo* with each feeding pattern for 4 weeks (Figs 3 and S4). The results suggest that the caloric intake during the inactive period with a 5-min RF, which has a phase shift effect on peripheral clocks but not on the central clock, would be an important causal factor of the time-specific depression-like behavior.

### Five-minute RF with HFD affects 5-HT levels and 5-HT metabolism in the hippocampus and striatum (Experiment 6)

**Figure 6:**
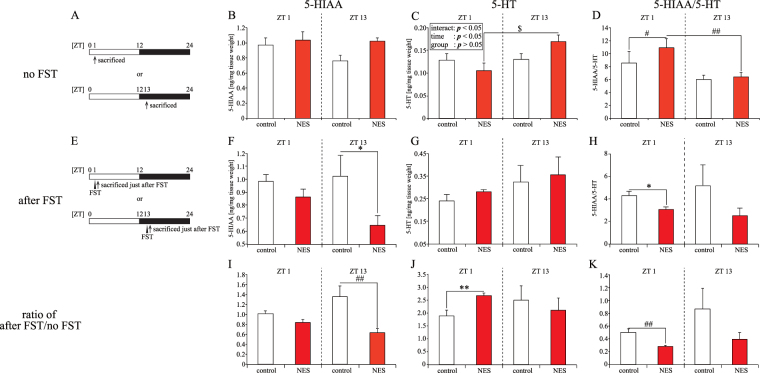
Effect of RF duration of 5-min with HFD at ZT 5 on 5-HT secretion and metabolism in the hippocampus at ZT 1 and 13 with and without FST (Experiment 6). (**A**) Sampling schedules. Mice were sacrificed at ZT 1 or 13 without FST, after mice were kept on each feeding condition for 4 weeks (no FST). (**B**–**D**) The 5-HIAA (**B**), 5-HT (**C**), and the metabolic rate (5-HIAA/5-HT; D) levels in the hippocampus at ZT 1 and 13. (**E**) Sampling schedules. Mice were sacrificed at ZT 1 or 13 just after the FST, after mice were kept on each feeding condition for 4 weeks (after FST). (**F**–**H**) The 5-HIAA (**F**), 5-HT (**G**), and the metabolic rate (H) levels in the hippocampus at ZT 1 and 13 just after the FST. (**I**–**K**) Stress reactivity of 5-HIAA (**I**), 5-HT (**J**), and metabolic rate (**K**) in the hippocampus. We calculated these levels by dividing each level after FST by the average with no FST. We show the results of two-way ANOVA in C. Data are presented as the mean ± SEM (*n* = 6–8). *p < 0.05, **p < 0.01 (Student’s *t*-test); ^#^*p* < 0.05, ^##^*p* < 0.01 (Mann-Whitney test); ^$^*p* < 0.05 (two-way ANOVA with Tukey’s multiple comparison test).

To understand the cause of the time-specific depression-like behavior in NES model mice, we examined the 5-HT levels and metabolic rate in the hippocampus and striatum using high performance liquid chromatography-electrochemical detection (HPLC-ECD) (Figs 6 and S10, Tables S1 and S2). Under conditions of no FST (Figs 6A and S10A), the 5-HT and 5-HIAA levels in the hippocampus and striatum, and the metabolic rate (5-HIAA/5-HT) in the striatum were similar in both groups (Figs 6B,C, and S10B–S10D). The 5-HT and metabolic rate levels in the NES model group showed significant differences between ZT 1 and 13, while the control group showed the same levels (Fig. 6C,D). Moreover, the 5-HIAA/5-HT level in the NES model group was increased compared to that in the control group at ZT 1 (Fig. 6D). A previous study reported an increased monoamine metabolic rate in mice showing depression-like behavior^31^. Thus, we examined the 5-HT, 5-HIAA, and 5-HIAA/5-HT levels in mice under stress. For this, we collected samples just after the FST (Figs 6E and S10E). The FST immobility time is shown in Fig. 4A. In the hippocampus, the 5-HIAA level decreased in the NES model group at ZT 13, and the 5-HIAA/5-HT level decreased in the NES model group at ZT 1 (Fig. 6F,H). The 5-HT levels in the hippocampus and the 5-HT, 5-HIAA, and 5-HIAA/5-HT levels in the striatum were similar in both groups (Figs 6G and S10F–S10H). To elucidate the net effect of stress on the 5-HT system, the after FST/no FST ratio was calculated (Figs 6I–K and S10I–S10K). The ratio of 5-HIAA in the hippocampus and striatum was decreased in the NES model group at ZT 13, and that of 5-HT in the hippocampus was increased in the NES model group at ZT 1. The ratio of 5-HIAA/5-HT in the hippocampus showed that a ratio less than 1 corresponded to decreased the 5-HT activity; this ratio was significantly decreased in the NES model group at ZT 1 (Figs 6I–K and S10I).

### Five-minute RF with HFD affects NE levels and NE metabolism in the hippocampus and striatum (Experiment 6)

**Figure 7:**
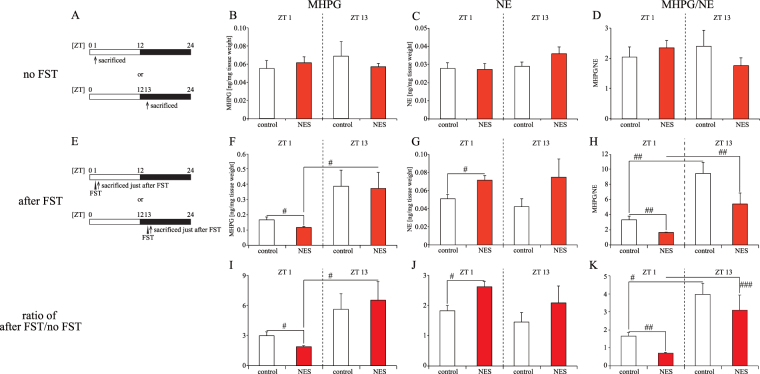
Effect of 5-min RF with HFD at ZT 5 on NE secretion and metabolism in the hippocampus at ZT 1 and 13 with and without FST (Experiment 6). (**A**) Sampling schedules. Mice were sacrificed at ZT 1 or 13 without FST, after mice were kept on each feeding condition for 4 weeks (no FST). (**B**–**D**) MHPG (**B**), NE (**C**), and the metabolic rate (MHPG/NE; **D**) levels in the hippocampus at ZT 1 and 13. (**E**) Sampling schedules. Mice were sacrificed at ZT 1 or 13 just after the FST, after mice were kept on each feeding condition for 4 weeks (after FST). (**F**-**H**) MHPG (**F**), NE (**G**), and the metabolic rate (**H**) levels in the hippocampus at ZT 1 and 13 after the FST. (**I**-**K**) Stress reactivity of MHPG (**I**), NE (**J**), and metabolic rate (**K**) in the hippocampus. We calculated these levels by dividing each level after FST by the average with no FST. Data are presented as the mean ± SEM (*n* = 6–8). ^#^*p* < 0.05, ^##^*p* < 0.01, ^###^*p* < 0.001 (Mann-Whitney test).

To understand the cause of the time-specific depression-like behavior in the NES model mice, we examined the NE levels and metabolic rate in the hippocampus and striatum (Figs 6 and S11, Tables S1 and S2). For no FST (Figs 7A and S11A), the NE, MHPG, and NE metabolic rate (MHPG/NE) levels in the hippocampus and striatum were similar in the control and NES model groups at ZT 1 and 13 (Figs 7B–D and S11B–S11D). After FST (Figs 7E and S11E), the hippocampus NE and MHPG levels in the NES model group were increased and decreased, respectively, compared with that in control group at ZT 1, but not at ZT 13 (Fig. 7F,G), and the hippocampus MHPG/NE level was also decreased in the NES model group at ZT 1 (Fig. 7H). In the striatum, there were no significant differences in these parameters between the control and NES model groups (Figure S11F–S11H). To elucidate the net effect of stress on the NE system, the after FST/no FST ratio was calculated (Figs 7I–K and S11I–S11K). Interestingly, this ratio exceeded 1, which indicated an increase of NE activity by acute stress. The change of NE, MHPG, and MHPG/NE in the hippocampus (Fig. 7I–K) were similar to those in groups after exposure to the FST (Fig. 7F–H). In the striatum, the ratio of MHPG at ZT1 in the NES model group was greater than that in the control group, and other parameters were similar in both groups (Figure S11I–S11K).

In the NES model group, a stressor at ZT 1 caused an increase in the 5-HT and NE secretion in the hippocampus, but decreased monoamine utilization and metabolism compared to those in the control group. Taken together, these results indicate that a disturbed feeding pattern may cause malfunction of the stress reactivity of 5-HT and NE at ZT 1 in the hippocampus, which may cause the time-specific depression-like behavior.

### Desipramine might decrease FST immobility time at ZT 1 induced by the NES feeding pattern (Experiment 7)

**Figure 8:**
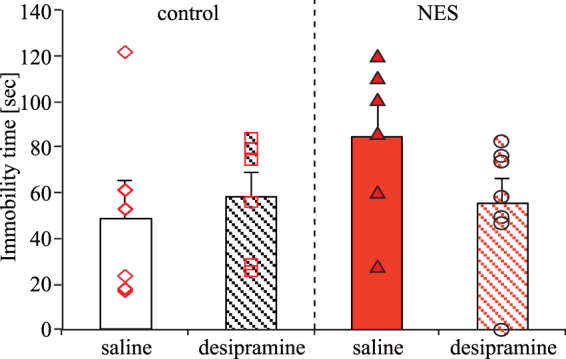
Effect of desipramine on FST immobility time induced by NES feeding condition (Experiment 7). The FST immobility time at ZT 1 after keeping mice to each feeding condition for 4 weeks. Each plot depicts individual data. Mice under each feeding condition were injected with saline or desipramine 30 min before the FST. Data are presented as the mean ± SEM (n = 6–7).

To examine whether the prolonged FST immobility time induced by the NES feeding condition was suppressed by desipramine treatment (10 mg/kg), we conducted the FST 30 min after desipramine or saline injection using the NES model group. Desipramine is a tricyclic antidepressant that has antidepressant-like effect on mice in the FST^44,45^. The FST immobility time in the NES-desipramine group was shorter than that in the NES-saline group, but there was not a significant difference (Fig. 8). This result indicated that the time-specific depression-like behavior induced by the NES feeding pattern may be improved by antidepressants, such as tricyclics.

## Discussion

The present study is the first demonstration of the relationship between disturbed feeding rhythm and time-specific depression-like behavior. The novel NES mouse model features a disturbed feeding rhythm similar to the eating pattern of patients with NES. Secondly, we demonstrate a gender-independent, time-specific, depression-like behavior caused by the disturbed feeding rhythm. Thirdly, the caloric intake during the inactive period was an important factor in the time-specific depression-like behavior; mice in the NES-5 min group, but not the NES-2 min group, showed the time-specific depression-like behavior. Fourthly, we suggest that the change of 5-HT and NE stress reactivity in the hippocampus is induced by the FST and these phenotypes might be one cause of the time-specific depression-like behavior. Lastly, we demonstrate that desipramine improves the time-specific depression-like behavior of some mice in the NES model group. Overall, this study indicates that the disturbed feeding rhythm could lead to the development of time-specific depression, and implicates the model as a good mouse model to further investigate the characteristics of patients with NES.

In this study, we first established the NES mouse model and confirmed its reproducibility and validity. In terms of feeding, the NES model group acquired approximately 25% of their total caloric intake during RF with HFD, displayed similar caloric intake between the active and inactive periods, and had a total caloric intake similar to that in the control group. A previous study showed that patients with NES consume more calories during the night (inactive period) than during the day (active period), although they had the same caloric intake per day as healthy subjects^46^. One of the core criteria for this disease is to have the consumption of at least 25% of the total calories after dinner^16^. In terms of locomotor activity, the NES model group was more active during the middle of the inactive period than the control group, and their duration of low locomotor activity was short and intermittent. In reality, patients with NES are characterized by frequent nocturnal awakening and experience difficulties in maintaining sleep^47^. In the present study, in terms of depression, the NES model group showed the depression-like behavior only at ZT 1. The mood of patients with NES declines during the evening and night, contrary to the typical pattern of depression^18^. The criteria for NES were established by observing the eating patterns in obese females^48^. Another study also reported male patients with NES^49^. In our study, we confirmed that the NES feeding increased body weight, affected locomotor activity rhythms, and prolonged the FST immobility time at ZT 1 using male and female mice. A recent study showed that circulating ovarian hormones could protect the integrity of daily rhythms in female mice during HFD feeding^50^. Moreover, we observed that body weight in the NES-ZT17-HFD group was similar to that in the control group. Accordingly, body weight and circadian clock system in the NES model group were more likely to be influenced by feeding rhythm, not by feeding with HFD itself. The collective results demonstrate that the NES mouse model based on the eating pattern in patients with NES is a very valid model of the human condition.

The FST immobility time and locomotor activity rhythm in the NES-ZT1 and NES-ZT9 groups were similar to those in the control group, although mice in the NES-ZT1 and NES-ZT9 groups ate HFD and their HFD calorie intakc was similar to that in the NES model group. These results suggest that RF at ZT 1 and ZT 9 are too early and late, respectively, to mimic the eating pattern of patients with NES.

We observed that a 5-min RF with HFD, which was associated with a disturbed feeding rhythm, for 4 weeks prolonged the FST immobility time only at ZT 1. We defined this phenotype as time-specific depression-like behavior. Feeding with HFD for more than 20 weeks can prolong immobility time in the FST^36^, and the Flinders Sensitive Line rat used as a genetic animal model of depression displays prolonged immobility time in the FST after consuming HFD for 8 weeks^51^. Taken together, these results indicate that feeding rhythm might be a more important contributor than dietary components to development of depression.

In this study, we observed that the FST immobility time at ZT 1 in the NES model group was longer than that in the control group, and that the TST immobility time at ZT1 in both groups was similar. A review paper indicated that the FST immobility corresponds to a state of behavioral despair, with the presumption that mice had not further desire to escape^52^, and that the TST immobility reflects an inescapable aversive stimulation^53^. Taken together, these results suggest that the NES feeding pattern might cause behavioral despair, rather than aversive stimulation, in mice.

When we examined the immobility time in the pre-FST and FST, we observed that the change of the pre-FST and FST immobility times were similar, but there were no significant differences between the control and NES model groups in the pre-FST immobility time. A previous study reported a longer FST immobility time than the pre-FST immobility time^54^. Taken together, these results indicate that the pre-FST and FST immobility times can be expressions of behavior despair, and that behavior despair in the FST is expressed more strongly than that in the pre-FST.

The present results reveal that the amount of calories consumed during the inactive period with a 5-min RF, which has the potential to shift the peripheral clocks, might be an important factor causing the time-specific depression-like behavior. Many studies in mice and humans indicate that restricted feeding affect the peripheral clocks and not the central clock^8–10,55^. In fact, presently RF with HFD at ZT 5 feeding advanced the peripheral clocks. Disruption of the relationship between the central and peripheral clocks induces depression-like behavior, because stress factors disrupt this relationship, which might result in disturbances in overt rhythmicity^56^. In humans, misalignment of biological and social time might be a risk factor for developing depression^57^, and circadian rhythm anomalies have been observed in seasonal affective disorder, bipolar disorder, and other mood disorders^58–60^. One study revealed that mice under abnormal LD cycles showed arrhythmicity and damped clock gene expression rhythm in the hippocampus together with prolonged the FST immobility time^61^. We could monitor PER2::LUC expression rhythm in peripheral clocks and central clock, but not in the hippocampus, and observed a phase shift of peripheral clocks in only the NES-5 min group. In addition, previous studies indicated the interaction of sleep quality with depression^62,63^. Locomotor activity during the middle of the inactive period in the NES-2 min group was increased compared to that in the control group, but the FST immobility time in the NES-2 min group was similar to that in the control group. Moreover, the FST immobility time in the NES-ZT5-ND group was similar to that in the control group, although sleep in the NES-ZT5-ND group was disturbed by RF with ND. Furthermore, the FST immobility time in the NES-ZT17-HFD group was similar to that in the control group, although they consumed HFD. In short, the time-specific depression-like behavior in the NES model group would be caused by disturbed feeding rhythm, not by repeated sleep disturbance and feeding with HFD. Conclusively, these results suggest that disturbed feeding rhythm is the cause of the difference among the central clock, environmental clock, and peripheral clocks, and that these differences might contribute to the development of the time-specific depression.

In this study, there were no significant differences in body weight between the control and NES model groups. However, we observed that the NES feeding pattern negated the differences in the RER levels between the inactive and active periods, suggesting that lipid beta-oxidation was decreased in the NES model group. In addition, the EE levels in the NES model group were higher than those in the control group, suggesting that the disturbed feeding rhythm might be cancelled by high energy consumption.

The disturbed feeding rhythm caused depression-like behavior only at ZT 1, which corresponded to evening and early night for human. Our previous study showed that sub-acute physical stress around ZT 1 causes internal desynchronization in the circadian clocks of peripheral tissues^7^. In short, physical stress around ZT 1 would cause abnormal reactions at least in the circadian clock system. Previous studies inconsistently showed that the FST can increase and/or decrease the 5-HT, 5-HIAA, NE, and MHPG levels in the hippocampus and the metabolic rate of both monoamines^64–67^. In the present study, the after FST/no FST ratio in the metabolic rates of both monoamines in the hippocampus in the NES model group were decreased compared to that in the control group at ZT 1, although the ratios of after FST/no FST in both monoamines in the hippocampus in the NES model group were increased. Compared to the control group, the synthesis of 5-HT and NE in the hippocampus in the NES model group was increased under acute stress at ZT 1, which was caused by the FST, but utilization and metabolism of these monoamines were not sufficiently increased under acute stress at ZT 1. Since behavioral change, such as the FST immobility time, may be related to the brain given the function of the striatum in the control of motion, we examined the monoamine change in the striatum. However, we observed that monoamines and their metabolism in the striatum were not changed in the NES model group. Taken together, these results suggest that a disturbed feeding rhythm causes the time-specific depression-like behavior that involves monoamine metabolism in the hippocampus, but not the striatum, and that the disturbed feeding rhythm might evoke malfunction and/or disuse of 5-HT and NE under stress, rather than synthesis of these monoamines in the hippocampus.

In this study, data from the NES model group indicated that phase shift of peripheral clocks and malfunction and/or disuse of 5-HT and NE in the hippocampus would be the cause of the time-specific depression-like behavior. We could not monitor the PER2::LUC bioluminescence rhythm in the hippocampus. However, the hippocampus PER2::LUC bioluminescence rhythm in the NES model group might indeed be shifted by the NES feeding pattern, because a previous study showed that RF during the inactive period caused a hippocampus PER2::LUC bioluminescence rhythm phase shift^68^. Other authors reported that RF induced phase shift in *Per2* expression rhythm and also in the expression rhythms of other clock genes^69^. Another study suggested that the change in expression of clock genes might contribute to the disturbances associated with depressive disorders^70^. Indeed, the expression levels of *Maoa* and *Comt*, which are enzymes catalyzing monoamine degradation, are regulated by clock genes and glucocorticoid^71^. Taken together, these reports suggest that the NES feeding pattern might shift the expression rhythm of clock genes in the hippocampus, and might change the expression levels of *Maoa* and *Comt*, leading to malfunction and/or disuse of 5-HT and NE in the hippocampus.

We revealed that the desipramine tricyclic shortened the FST immobility time of some (less than half) of the mice in the NES model group. In humans, the depressive symptom is improved in half of NES patients by drugs including SSRIs and Topiramate^34,35^. Our NES model phenotype was similar to the phenotype in NES patients treated with SSRIs. However, we still do not understand why the effect of desipramine on depression-like behavior depended on individual mice.

Some reviews suggested that leptin levels in patients with NES are similar to those in the healthy group^72,73^. On the other hand, another study demonstrated delayed leptin secretion rhythm in patients with NES^74^. In this study, we observed that the serum leptin levels at ZT 1 and 13 in the NES model group were similar to those in the control group. Moreover, a previous study showed that the amplitude of cortisol secretion rhythm was decreased in patients with NES compared to the healthy group^74^. Similar to human observations, the NES model group presently had a low ratio of serum corticosterone at ZT 13 to that at ZT 1, suggesting the low amplitude of corticosterone release rhythm. Further experiments are needed to clarify the relationship between the NES feeding pattern and hormone secretion rhythms throughout the day.

In this paper, we established a new NES mouse model by focusing on the NES feeding pattern. The model demonstrated the relationship between the disturbed feeding rhythm and time-specific depression-like behavior for the first time. Moreover, our results demonstrated that the malfunction of hippocampus 5-HT and NE under stress can be induced by disturbed feeding rhythm, and that the caloric intake during the inactive period might be an important contributing factor to the development of time-specific depression. These experimental results might be widely applicable for people, even though we established the NES model focused on NES feeding pattern, since many people in modern societies work and play during the night. We suggest that using this model may help to understand the characteristics of NES patients, which could lead to a remedy for NES. The reason is that studies of treating for NES have been even in primary, although studies have been conducted^49^.

## Materials and Methods

### Animals

Eight-week-old, ICR mice and PER2::LUC knock-in mice were housed in an animal room that was maintained at 22 ± 2 °C and humidity 60 ± 5%, with a 12-h light/12-h dark cycle (lights-on from 08:00 to 20:00). ZT 0 and 12 were defined as the lights-on and -off time, respectively. We used mainly female mice in the study, because the criteria for NES were established by observing the eating patterns in a group of obese females^48^. However, we compared male mice with female mice in the experiment 5 (Figure S9). The PER2::LUC knock-in mice were used in the experiment 3 and ICR mice were used in all other experiments. Heterozygous PER2::LUC knock-in mice were bred in our laboratory and housed in the animal room as previously described^40^. They were provided with ND (EF; Oriental Yeast Co. Ltd., Japan) and water *ad libitum* before experiments. The procedures conformed to the “Fundamental Guidelines for Proper Conduct of Animal Experiments and Related Activities in Academic Research Institutions” (published by the Ministry of Education, Culture, Sports, Science and Technology, Japan) and were approved by the Committee for Animal Experimentation at Waseda University (permission #2016-A065).

### Diets

We supplied mice with ND and/or HFD during the experiments. The HFD consisted of the AIN-93M formula (Oriental Yeast Co. Ltd., Tokyo, Japan) combined with lard oil (Sigma-Aldrich, St. Louis, MO, USA) in a ratio of 4:1. For the ND, 10% of the calories were from fat. In the HFD, 44.9% of the calories were from fat.

### Feeding schedule

We divided the mice into the control and NES model groups. Each mouse in both groups was kept in an individual cage with ND and water available *ad libitum*. In addition, in the NES model group, access to HFD was restricted starting at ZT 5. The feeding pattern in the NES model group imitated the eating pattern in patients with NES. Body weight and food volume intake were measured every week. Food volume intake was expressed as kcal/mouse/day.

### Locomotor activity analysis

Each mouse was housed in an individual cage during experiments. Locomotor activity was measured with a model SE-10 infrared radiation sensor (Akizuki Denshi Tsusho Co. Ltd., Tokyo, Japan) and analyzed using CLOCKLAB software (Actimetrics, Wilmette, IL, USA). Locomotor activity was measured as the number of sensor counts every 30 min. The percentage of locomotor activity in each time period was calculated using the following formula: (counts for 1 h/counts for 1 day) × 100.

### *In vivo* monitoring protocol of peripheral clocks and data analysis

*In vivo* monitoring and data analysis were carried out as described previously^40^. Briefly, mice were anesthetized with isoflurane (Mylan Inc., Tokyo, Japan) and concentrated oxygen (SO-005B; Sanyo Electronic Industries Co. Ltd., Okayama, Japan) in an *in vivo* kinetics imaging system (Caliper Life Sciences, Hopkinton, MA, USA). While mice were under anesthesia, they were injected subcutaneously on the back and near the neck with D-luciferin potassium salt (Promega, Madison, WI, USA) at a dose of 15 mg/kg body weight. Images were taken 8 and 10 min after D-luciferin injection in the dorsal-up position for the kidney and in the ventral-up position for the liver and the submandibular gland, respectively. Each image used an exposure time of 1-min. Images were obtained six times per day at 4-h intervals, using the same mice. Mice were returned to their home cages after each imaging procedure and recovered quickly from isoflurane anesthesia. Bioluminescence emitted from the kidney and the liver was calculated automatically using Living Image 3.2 software (Caliper Life Sciences). For these organs, the average photon/min value of the data from the six time points was designated as 100%, and the bioluminescent rhythm for the entire day was expressed as a percentage. The peak phase of the normalized percent data was determined using the single cosinor procedure program (Acro.exe, version 3.5)^75^. In a previous study, these procedures did not affect the peripheral clocks^40^.

### Forced swimming test (FST)

The method of the FST was developed previously^38^ and was modified in subsequent studies^76–79^. Briefly, mice were placed in a polymethylpentene cylinder (diameter 137 mm; height 200 mm) filled with water at a temperature of approximately 25 °C to a depth of approximately 150 mm for 15 min as a pre-test (pre-FST). After 24 h, mice were forced to swim again for 6 min and were recorded the entire time using a video camera. Immobility time was measured for the last 4 min of the swim period by observers who had no information about experimental conditions. Immobility was defined as when mice stopped swimming and floated on the water.

### High performance liquid chromatography-electrochemical detection (HPLC-ECD)

We measured the levels of 5-HT, 5-HIAA, NE, and MHPG in the hippocampus and striatum by HPLC-ECD (HTEC 500; Eicom, Kyoto, Japan) following a previous protocol^6,7^. In the experiment 6, mice were maintained at each feeding schedule for 4 weeks before sacrifice. At ZT 1 and 13, mice were sacrificed with isoflurane without or after the FST, and the hippocampus and striatum were removed for HPLC-ECD. Samples for 5-HT, 5-HIAA, NE, and MHPG measurement received 0.2 M perchloric acid (including 100 μM EDTA·2Na) containing 20 ng of isoproterenol. Samples were homogenized using a micro-homogenizer before being centrifuged at 15,000 × g at 4 °C for 15 min. The supernatant for each sample was collected and filtered using a 0.45 μm filter. The quantity of monoamine in each 20 μL sample was measured by HPLC-ECD with the following conditions: 85% of the transfer phase was composed of 0.1 M acetate citric acid buffer (pH 3.5), including 5 mg/L EDTA·2 Na, 190 mg/L 1-octanesulfonic acid sodium salt, and 15% methanol. The velocity of the flow was 500 μL/min. The column temperature was 25 °C and the applied voltage was + 750 mV versus Ag/AgCl. The data were evaluated by EPC-300 software (Eicom). Dopamine could not be detected in the hippocampus.

### Drug

Desipramine (10 mg/kg, Sigma-Aldrich) was administered 30 min prior to the FST and was injected intraperitoneally. Mice in the saline group were injected with saline.

### Statistical analyses

All values are expressed as mean ± standard error of the mean (SEM). Statistical analyses were calculated using GraphPad Prism version 6.03 (GraphPad Software, San Diego, CA, USA). We ascertained whether data showed normal or non-normal distribution, and equal or biased variation was assessed by D’Agostino-Pearson normality test/Kolmogorov-Smirnovt test/one-sample *t*-test, and *F*-value test/Bartlett test, respectively. Parametric analysis was calculated using Student’s *t*-test or one-way ANOVA with Tukey’s multiple comparison test, and non-parametric analysis was conducted using the Mann-Whitney test. We performed two-way ANOVA with Tukey’s multiple comparison test only when the data showed normal distribution and equal variation. Conversely, we performed Student’s *t*-test, one-way ANOVA with Tukey’s multiple comparison test, Mann-Whitney test, or Kruskal-Wallis test with Dunn’s multiple comparison test when the data showed non-normal distribution or biased variation. Detailed data for all statistical analyses of all results are shown in Tables S1 and S2.

## Electronic supplementary material

supplemental information
