# Supplementary material for: Night eating model shows time-specific depression-like behavior in the forced swimming test
Source: Sci Rep. 2018 Jan 18;8:1081. doi: 10.1038/s41598-018-19433-8 (PMC5773531; doi:10.1038/s41598-018-19433-8)
Supplement: Supplementary file 1 — supplemental information [file 41598_2018_19433_MOESM1_ESM.pdf]

Supplementary Information

Night eating model shows time-specific depression-like behavior in the forced swimming test

Atsushi Haraguchi#, Miyabi Fukuzawa#, Shiho Iwami, Yutaro Nishimura, Hiroaki Motohashi, Yu Tahara, and Shigenobu Shibata*

Laboratory of Physiology and Pharmacology, School of Advanced Science and Engineering, Waseda University, Tokyo, Japan

#These authors contributed equally to this work.
*Corresponding author: Shigenobu Shibata
Laboratory of Physiology and Pharmacology, School of Advanced Science and Engineering, Waseda University, Shinjuku-ku, Tokyo 162-8480, Japan
Tel: +81-3-5369-7318, E-mail address: shibatas@waseda.jp

Supplemental methods

Energy expenditure

We measured energy expenditure (EE) and the respiratory exchange ratio (RER) using an Oxymax Lab Animal Monitoring System (Columbus Instruments, Columbus, OH, USA). Mice were housed individually for 2 days in a chamber (190 × 100 × 120 mm) under each feeding schedule. Exhausted air from each chamber was sampled and passed through O_2 and CO_2 sensors for measurements of the amounts of O_2 and CO_2. Subsequently, O_2 consumption (VO_2) and CO_2 production (VCO_2) were calculated. The RER was calculated as the ratio of VCO_2 to VO_2. The EE was calculated using followed formula; $(3.185 + 1.232 \times RER) \times VCO_2$.

Measurement of bioluminescence from *ex vivo* cultures of liver and SCN

PER2::LUC mice were euthanized at ZT 7 for the measurement of bioluminescence rhythmicity in liver and SCN. The liver was rapidly removed from each euthanized mouse and placed in ice-cold Hanks' balanced salt solution (HBSS; pH 7.2; Sigma-Aldrich, St. Louis, MO, USA). Four small pieces were separated from the liver and explanted into a 35 mm Petri dish (AGC Techno Glass Co. Ltd., Tokyo, Japan). Slices 300 µm in thickness that contained SCN were obtained using a DTK-1500 vibratome (D.S.K., Kyoto, Japan). Each slice was placed on a membrane (0.4 µm, 30 mm in diameter; Millicell cell culture insert; Millipore, Billerica, MA, USA) in a 35 mm Petri dish. All dishes were sealed with parafilm (Sigma-Aldrich) and cultured in 2.0 mL Dulbecco's modified Eagle's medium (DMEM; Invitrogen, Carlsbad, CA, USA) additionally including NaHCO3 (2.7 mM), HEPES (10 mM), insulin (5 µg/mL; Sigma-Aldrich), kanamycin (20 mg/L; Sigma-Aldrich), putrescine (100 µM; Sigma-Aldrich), progesterone (20 nM; Sigma-Aldrich), human transferrin (100 µg/mL; Sigma-Aldrich), sodium selenite (30 nM; Sigma-Aldrich), and D-luciferin potassium salt (0.1 mM). All dishes were incubated at 37 °C, and the bioluminescence was monitored for 1 min at each 10 min interval with a dish-type luminometer.

Tail suspension test (TST)

Mice were each suspended from a bar 50 cm above the floor by the tail using adhesive tape placed approximately 1 cm from the tip of the tail. Mice were suspended for 6 min and were recorded the entire time by a video camera. The TST immobility time was measured for the last 4 min of the 6 min period by observers who had no information about experimental conditions.

Collection of blood for hormone analysis

Mice were maintained under each feeding condition for more than 4 weeks before blood samples were collected from the vein tail at ZT 1. After 7 days interval, blood samples from the vein tail were collected at ZT 13 from the same mice. The blood was allowed to clot and clots were removed from each sample by centrifugation at 3000 rpm for 30 min at 4 °C. Serum aliquots (400-600 mL) were collected and stored in sealable polypropylene micro-centrifuge tubes at -80 °C for subsequent analysis.

Serum corticosterone and leptin assay

Serum concentrations of corticosterone and leptin in mice were determined using enzyme-linked immunosorbent assay (ELISA) kits (ASSAYPRO LLC, St. Charles, MO, USA; and R&D Systems, Ind., Tokyo, Japan) according to the manufacturers' protocols. Detectability was within the calibration limits of the assay.

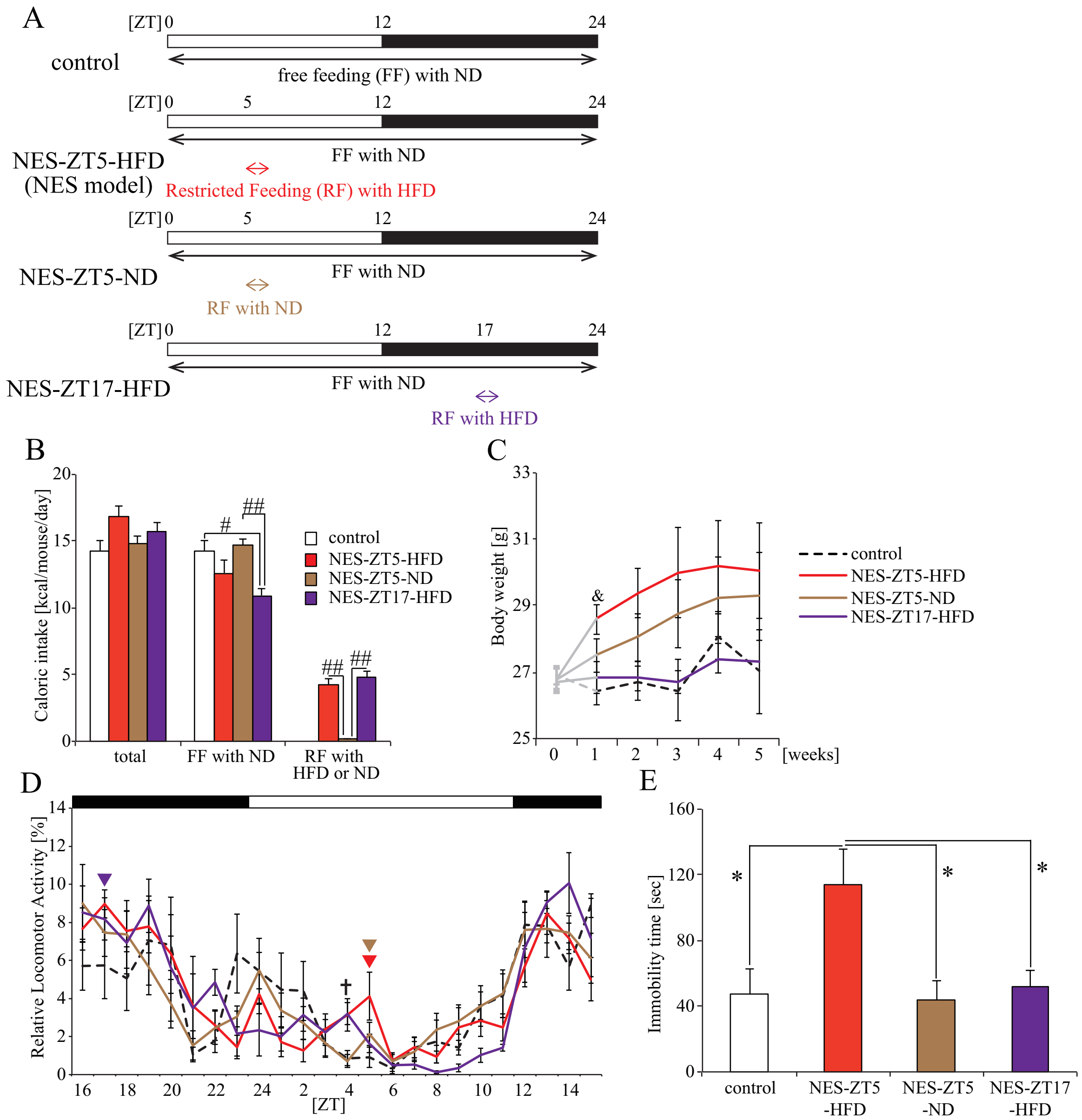

Supplemental figure S1. Effect of 5-min RF with HFD or ND at ZT 5 or 17 on locomotor activity, body weight, caloric intake, and FST immobility time (Experiment 1 and 4)

(A) Diagram of the experimental schedule. (B) Average caloric intake of total, FF with ND, and RF with HFD or ND under each feeding condition. (C) Increase in body weight. The gray lines indicate under-habituation. (D) Relative locomotor activity rhythm under each feeding condition. Red and purple triangles represent the start of RF with HFD, and brown triangle represents the start of RF with ND. Open and closed bars indicate light and dark period, respectively. (E) FST immobility time at ZT 1 after keeping mice on each feeding condition for 4 weeks. Data are presented as the mean ± SEM (n = 7). * $p < 0.05$ (One-way ANOVA with Tukey's multiple comparison test). ✝ $p < 0.05$ vs. the NES-ZT5-ND group (One-way ANOVA with Tukey's multiple comparison test). # $p < 0.05$, ## $p < 0.01$ (Kruskal-Wallis test with Dunn's multiple comparison test).

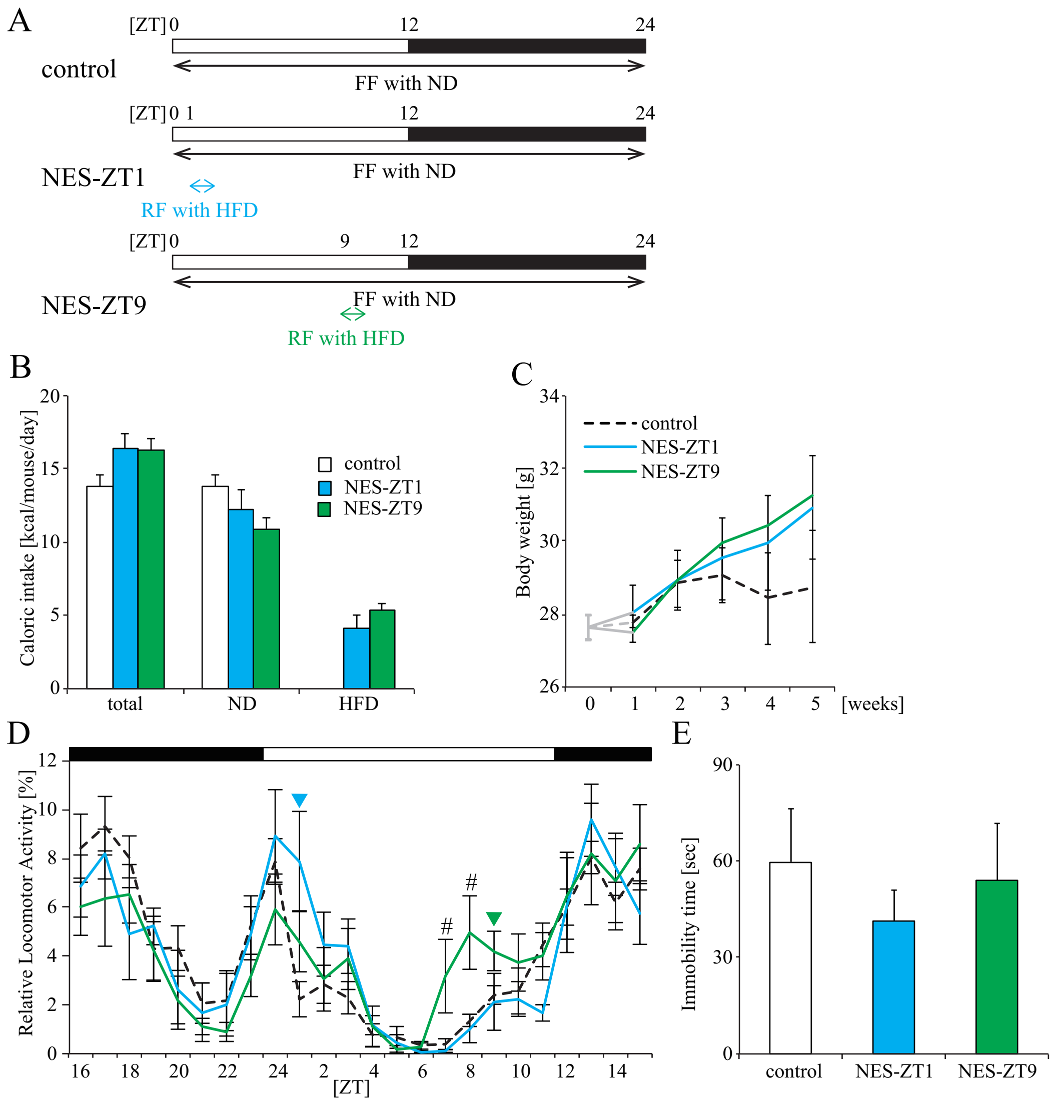

Supplemental figure S2. Effect of 5-min RF with HFD at ZT 1 and 9 on locomotor activity, body weight, caloric intake, and FST immobility time (Experiment 1 and 4)

(A) Diagram of the experimental schedule. (B) Average caloric intake of total, ND, and HFD under each feeding condition. (C) Increase in body weight. The gray lines indicate under-habituation. (D) Relative locomotor activity rhythm with a 5-min RF with HFD at ZT 1 and 9. Blue and green triangles represent the start of RF. Open and closed bars indicate light and dark period, respectively. (E) FST immobility time at ZT 1 after keeping mice on each feeding condition for 4 weeks. Data are presented as the mean ± SEM (*n* = 7). # $p < 0.05$ vs. the NES-ZT1 group (Kruskal-Wallis test with Dunn's multiple comparison test).

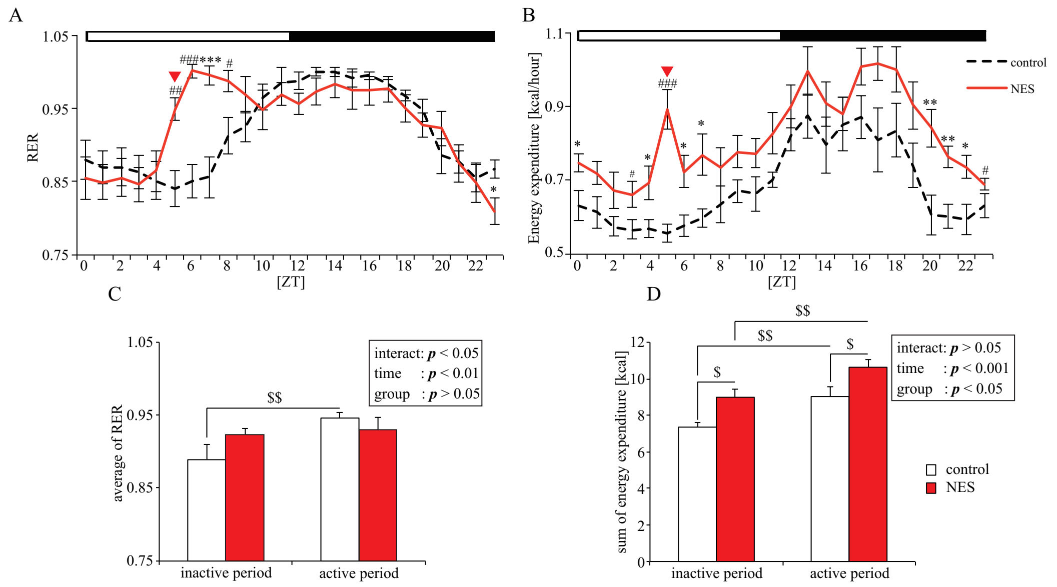

Supplemental figure S3. Effect of 5-min RF with HFD at ZT 5 on daily respiratory exchange ratio and energy expenditure rhythm (Experiment 2)

(A and B) The daily respiratory exchange ratio (RER) rhythm (A) and the daily energy expenditure (EE) rhythm (B). Red triangles represent the start of RF. (C) Average of the RER in the inactive and active periods. (D) Sum of the EE in the inactive and active periods. Data are presented as mean ± SEM (n = 8). We show the results of two-way ANOVA in C and D. * $p < 0.05$, ** $p < 0.01$, *** $p < 0.001$ vs. the control group (Student's *t*-test). # $p < 0.05$, ## $p < 0.01$, ### $p < 0.001$ vs. the control group (Mann-Whitney test). \$ $p < 0.05$, \$\$ $p < 0.01$ (two-way ANOVA with Tukey's multiple comparison test).

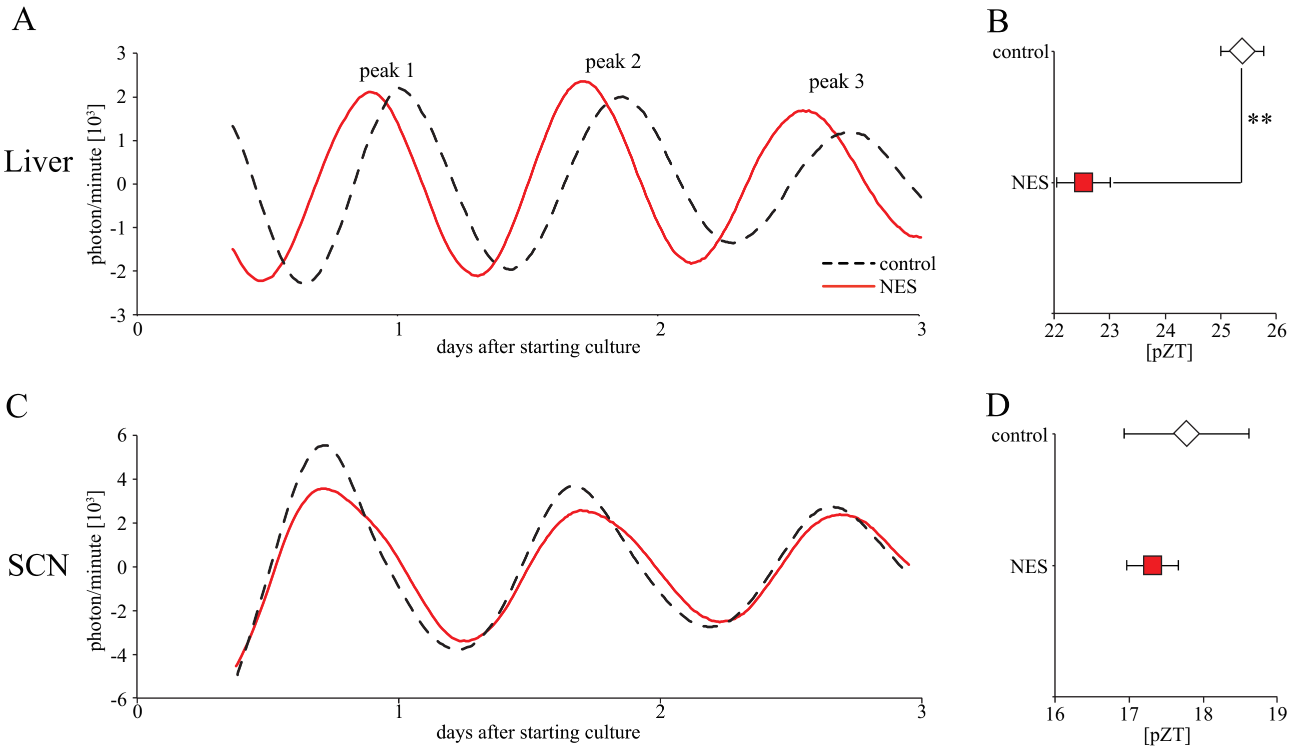

Supplemental figure S4. Effect of 5-min RF with HFD at ZT 5 on PER2 expression rhythms in SCN and liver (Experiment 3)

(A) Representative de-trended data of PER2::LUC bioluminescence rhythm in the liver. (B) Peak 1 phase of PER2::LUC bioluminescence rhythm in the liver. The horizontal axis indicates projected ZT (pZT) at the peak 1 of the PER2::LUC bioluminescence rhythm. (C) Representative de-trended data for PER2::LUC bioluminescence rhythm in SCN. (D) Peak 1 phase of PER2::LUC bioluminescence rhythm in SCN. Data are presented as mean ± SEM (control group, n = 5; NES groups, n = 7). ** $p < 0.01$ (Student's t-test).

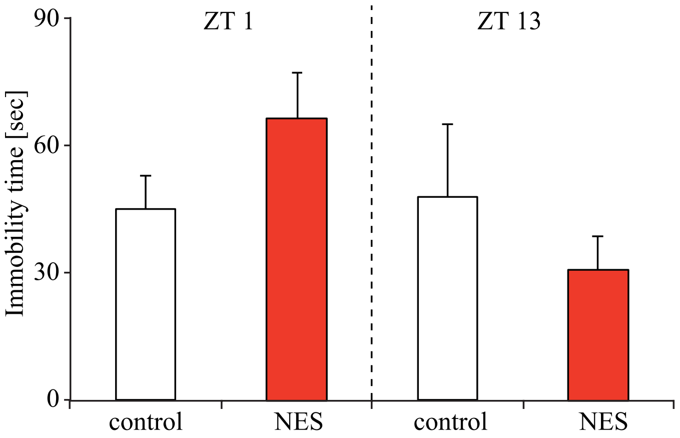

Supplemental figure S5. Effect of 5-min RF with HFD at ZT 5 on immobility time at ZT 1 in the pre-FST (Experiment 4)

Immobility time at ZT 1 and 13 in the pre-FST after keeping mice on each feeding regimen for 4 weeks. Data are presented as the mean ± SEM (control group, *n* = 6; NES groups, *n* = 7).

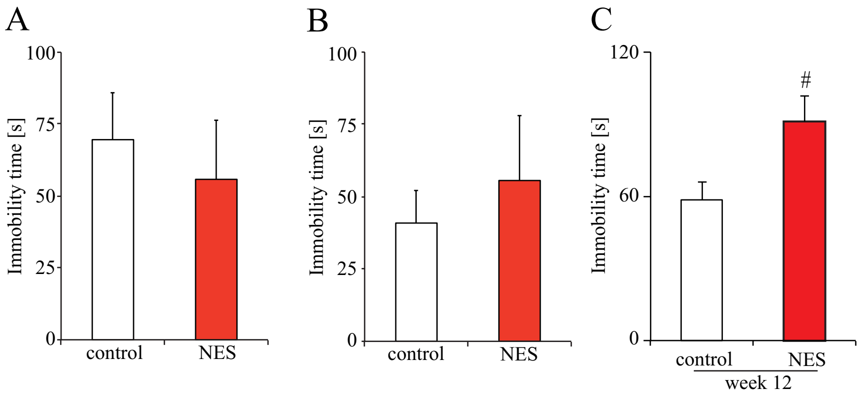

Supplemental figure S6. Effect of 5-min RF with HFD at ZT 5 on first FST immobility time (Experiment 4)

(A and B) FST immobility time at ZT 9 (A) and 17 (B) after keeping mice on each feeding regimen for 4 weeks. Data are presented as the mean ± SEM (n = 7). (C) FST immobility time at ZT 1, after subjecting mice to each feeding condition for 12 weeks (n = 8). # $p < 0.05$ (Mann-Whitney test).

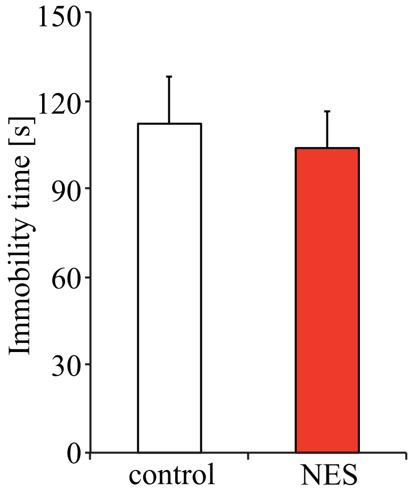

Supplemental figure S7. Effect of 5-min RF with HFD at ZT 5 on immobility time at ZT 1 in the tail suspension test (Experiment 4)

Immobility time at ZT 1 in the tail suspension test after keeping mice on each feeding regimen for 4 weeks. Data are presented as the mean ± SEM (*n* = 7).

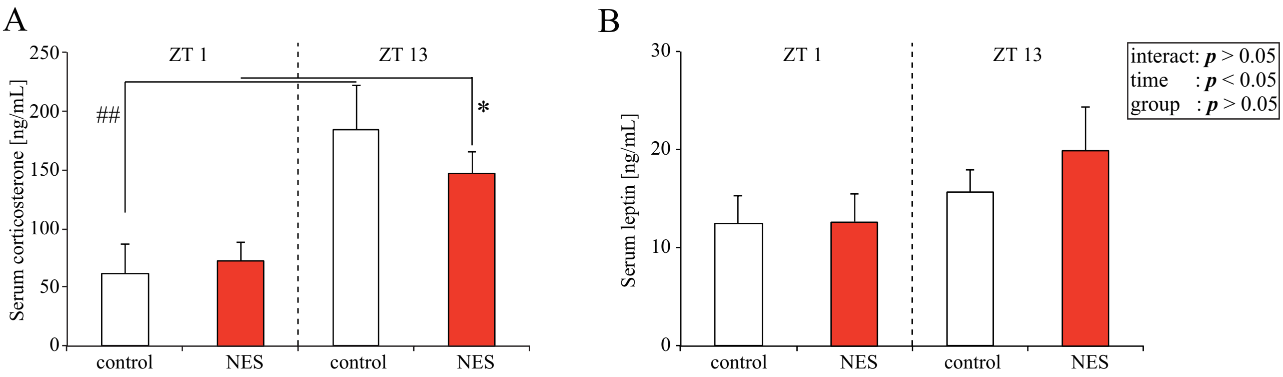

Supplemental figure S8. Effect of 5-min RF with HFD at ZT 5 on serum corticosterone and leptin at ZT 1 and 13 (Experiment 4)

(A) Serum corticosterone at ZT 1 and 13 after keeping mice on each feeding regimen for more than 4 weeks. (B) Serum leptin at ZT 1 and 13 after keeping mice on each feeding regimen for more than 4 weeks. We show the results of two-way ANOVA in B. Data are presented as the mean ± SEM (the control group, n = 10; the NES groups, n = 13). * $p < 0.05$ (paired t-test), ## $p < 0.01$ (Wilcoxon signed-rank test).

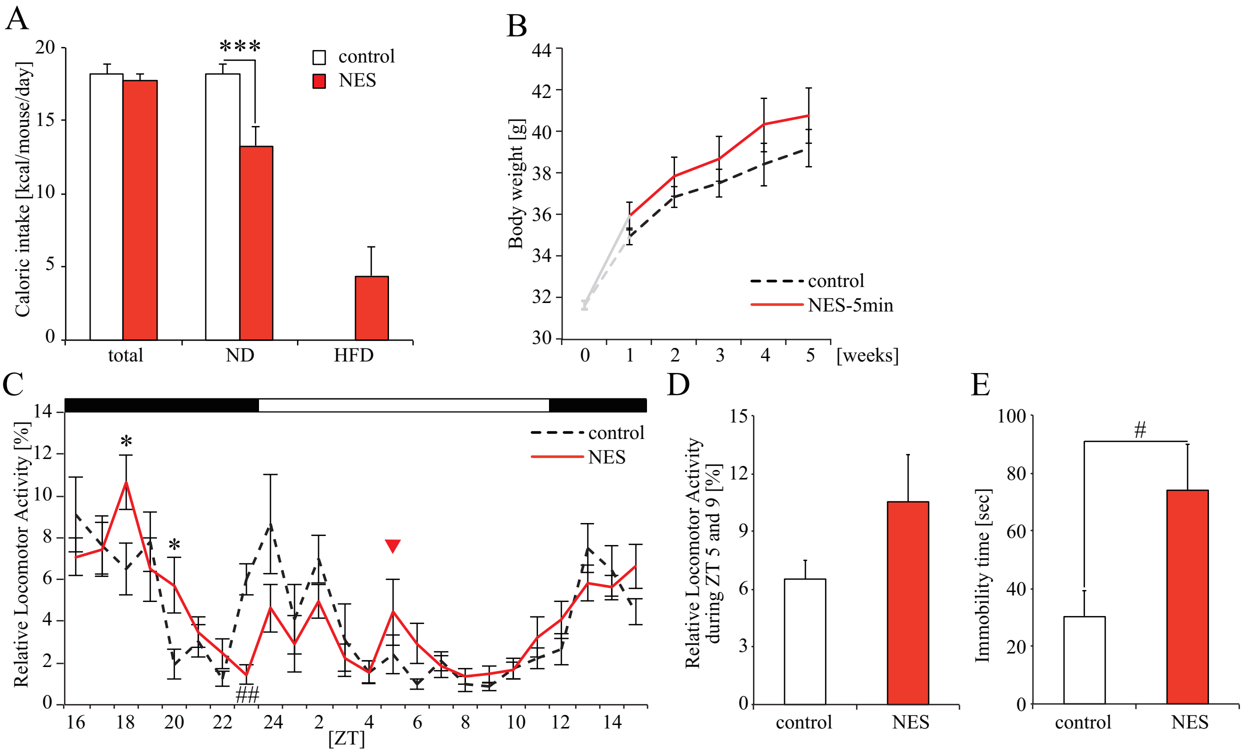

Supplemental figure S9. Effect of 5-min RF with HFD at ZT 5 on locomotor activity, body weight, caloric intake, and FST immobility time using male mice (Experiment 5)

(A) Average caloric intake of total, ND, and HFD under each feeding regimen. (B) Increase in body weight. The gray lines indicate under-habituation. (C) Relative locomotor activity rhythm with a 5-min RF with HFD at ZT 5. Red triangle represents the start of RF. Open and closed bars indicate light and dark period, respectively. (D) Relative locomotor activity during ZT 5 and 9. (E) FST immobility time at ZT 1 after keeping mice on each feeding regimen for 4 weeks. Data are presented as the mean ± SEM (n = 8). * $p < 0.05$, *** $p < 0.001$ (Student's t-test). # $p < 0.05$, ## $p < 0.01$ (Mann-Whitney test).

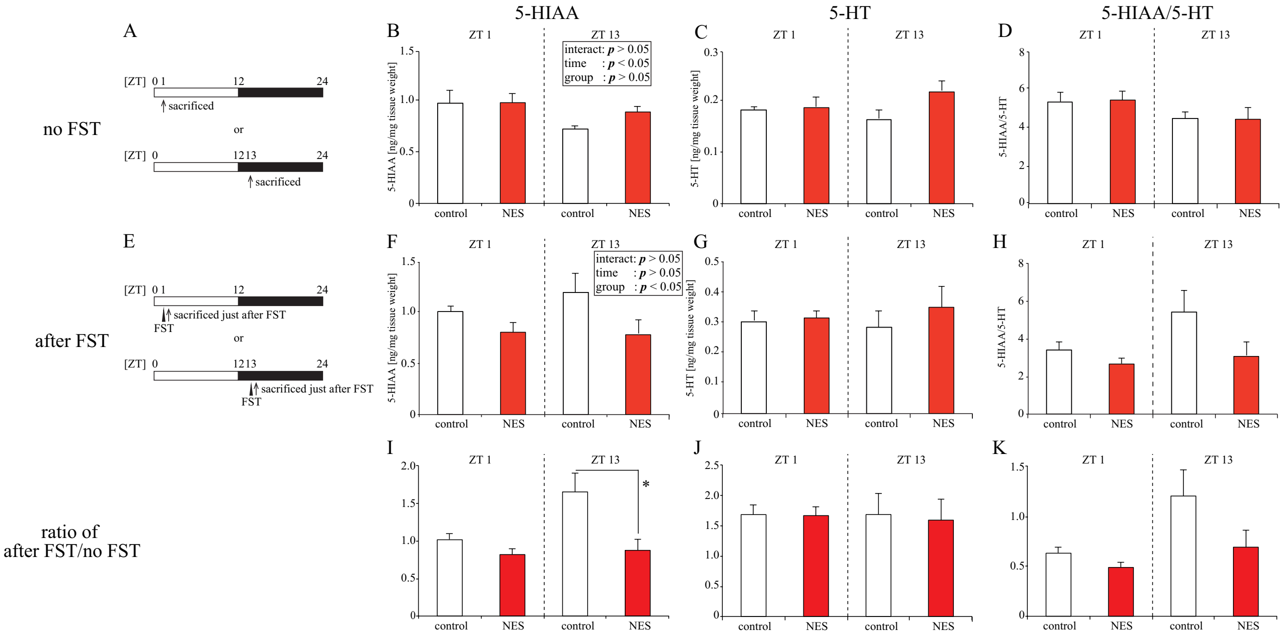

Supplemental figure S10. Effect of 5-min RF with HFD at ZT 5 on 5-HT secretion and metabolism in the striatum at ZT 1 and 13 with and without the FST (Experiment 6)

(A) Sampling schedules for no FST. Mice were sacrificed at ZT 1 or 13 without the FST, after mice were kept under each feeding regimen for 4 weeks (no FST). (B-D) 5-HIAA (B), 5-HT (C), and the metabolic rate (5-HIAA/5-HT; D) levels in the striatum at ZT 1 and 13. (E) Sampling schedules for after FST. Mice were sacrificed at ZT 1 or 13 just after the FST, after mice were kept on each feeding regimen for 4 weeks (after FST). (F-H) 5-HIAA (F), 5-HT (G), and the metabolic rate (H) levels in the striatum at ZT 1 and 13 just after the FST. (I-K) Stress reactivity of 5-HIAA (I), 5-HT (J), and metabolic rate (K) in the striatum. We calculated these levels by dividing each level after FST by the average with no FST. We showed the results of two-way ANOVA in B and F. Data are presented as the mean ± SEM (n = 6-8). * $p < 0.05$ (Student's t-test).

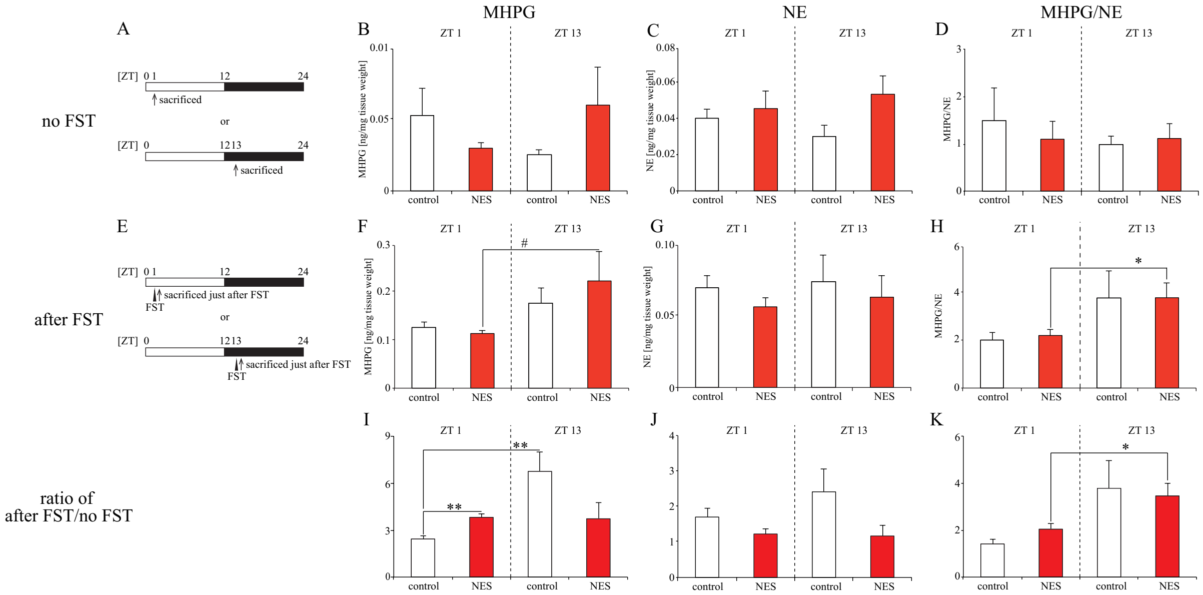

Supplemental figure S11. Effect of 5-min RF with HFD at ZT 5 on NE secretion and metabolism in the striatum at ZT 1 and 13 with and without the FST (Experiment 6)

(A) Sampling schedules for no FST. Mice were sacrificed at ZT 1 or 13 without the FST, after mice were kept under each feeding regimen for 4 weeks (no FST). (B-D) MHPG (B), NE (C), and the metabolic rate (MHPG/NE; D) levels in the striatum at ZT 1 and 13. (E) Sampling schedules for after FST. Mice were sacrificed at ZT 1 or 13 just after the FST, after mice were kept on each feeding regimen for 4 weeks (after FST). (F-H) MHPG (F), NE (G), and the metabolic rate (H) levels in the striatum at ZT 1 and 13 after the FST. (I-K) Stress reactivity of MHPG (I), NE (J), and metabolic rate (K) in the striatum. We calculated these levels by dividing each level after FST by the average with no FST. Data are presented as the mean ± SEM (n = 6-8). * $p < 0.05$, ** $p < 0.01$ (Student's *t*-test), # $p < 0.05$ (Mann-Whitney test).

Supplemental table S1. The results of 2-way ANOVA

Figure No.	group	n	distribution		main effect A				main effect B				interact effect		
				variation	factor	F	degree of freedom	p value	factor	F	degree of freedom	p value	F	degree of freedom	p value
4A	control	6	normal	equal	time	F (1, 22) = 4.037	1	0.057	feeding schedule	F (1, 22) = 1.472	1	0.238	F (1, 22) = 8.080	1	< 0.01
	NES	7	normal												
6C	control	8	normal	equal	time	F (1, 28) = 4.905	1	< 0.05	feeding schedule	F (1, 28) = 0.336	1	0.567	F (1, 28) = 4.495	1	< 0.05
	NES	8	normal												
7C	control	8	normal	equal	time	F (1, 28) = 2.288	1	0.142	feeding schedule	F (1, 28) = 1.046	1	0.315	F (1, 28) = 1.380	1	0.25
	NES	8	normal												
8	control	6	normal	equal	feeding schedule	F (1, 21) = 1.559	1	0.157	drug	F (1, 21) = 0.587	1	0.452	F (1, 21) = 2.159	1	0.157
	NES	6 or 7	normal												
S3C	control	8	normal	equal	time	F (1, 14) = 9.767	1	< 0.01	feeding schedule	F (1, 14) = 0.223	1	0.644	F (1, 14) = 6.096	1	< 0.05
	NES	8	normal												
S3D	control	8	normal	equal	time	F (1, 14) = 39.57	1	< 0.001	feeding schedule	F (1, 14) = 8.256	1	< 0.05	F (1, 14) = 0.004	1	0.9536
	NES	8	normal												
S5	control	6	normal	equal	time	F (1, 22) = 2.061	1	0.165	feeding schedule	F (1, 22) = 0.037	1	0.849	F (1, 22) = 2.943	1	0.1
	NES	7	normal												
S8B	control	10	normal	equal	time	F (1, 21) = 4.859	1	< 0.05	drug	F (1, 21) = 0.272	1	0.607	F (1, 21) = 0.729	1	0.403
	NES	13	normal												
S10B	control	8	normal	equal	time	F (1, 28) = 4.334	1	< 0.05	feeding schedule	F (1, 28) = 1.139	1	0.295	F (1, 28) = 1.028	1	0.319
	NES	8	normal												
S10F	control	6	normal	equal	time	F (1, 22) = 0.426	1	0.521	feeding schedule	F (1, 22) = 5.551	1	< 0.05	F (1, 22) = 0.714	1	0.407
	NES	7	normal												
S11C	control	8	normal	equal	time	F (1, 28) = 0.011	1	0.917	feeding schedule	F (1, 28) = 2.856	1	0.102	F (1, 28) = 1.109	1	0.301
	NES	8	normal												

Supplemental table S2. The results of statistical analysis on other results

Figure No.	group	n	distribution	variation: all	variation: each group	factor	test	F	degree of freedom	p value
1B-60min	control	6	non-normal			time	Friedman test			< 0.001
	NES	8	non-normal			time	Friedman test			< 0.001
1B-30min	control	6	non-normal			time	Friedman test			< 0.001
	NES	8	non-normal			time	Friedman test			< 0.001
1B-15min	control	6	non-normal			time	Friedman test			< 0.001
	NES	8	non-normal			time	Friedman test			< 0.001
1B-5min	control	6	non-normal			time	Friedman test			< 0.001
	NES	8	non-normal			time	Friedman test			< 0.001
1B-1min	control	6	non-normal			time	Friedman test			< 0.001
	NES	8	non-normal			time	Friedman test			< 0.001
1C	control	6	normal		equal	duration	repeated One-way ANOVA	F (2.181, 10.90) = 3.022	4	0.379
	NES	8	non-normal			duration	Friedman test			< 0.001
1D	control	6	normal		equal	week	repeated One-way ANOVA	F (1.951, 9.757) = 19.73	9	< 0.001
	NES	8	non-normal			week	Friedman test			< 0.001
1E	control	6	normal	biased	biased	week	Friedman test			< 0.01
	NES	8	normal		biased	week	Friedman test			< 0.001
1F	control	6	normal	biased	biased	week	Friedman test			< 0.01
	NES	8	normal		equal	week	repeated One-way ANOVA	F (3.484, 24.39) = 15.25	10	< 0.001
1G	NES	8	non-normal			week	Friedman test			< 0.001
2B	control	8	non-normal			week	Friedman test			0.141
	NES	8	normal			week	repeated One-way ANOVA	F (2.967, 20.77) = 3.022	11	0.053
2C	control	8	non-normal			week	Friedman test			0.141
	NES	8	non-normal			week	Friedman test			< 0.05
2D	NES	8	non-normal			week	Friedman test			< 0.001

2E	control	8	non-normal			time	Friedman test			< 0.001
	NES	8	non-normal			time	Friedman test			< 0.001
2F	control	8	non-normal			week	Friedman test			< 0.001
	NES	8	normal		equal	week	repeated One-way ANOVA	F (3.221, 22.55) = 15.99	11	< 0.001
2G	control	8	non-normal			feeding schedule	Mann Whitney test			< 0.05
	NES	8	normal							
2H	control	8	normal	biased	biased	time	Wilcoxon signed-rank test			< 0.01
	NES	8	normal		equal	time	paired *t*-test			0.561
3B	control	6	normal	biased	biased	time	Friedman test			< 0.001
	NES-5min	5	normal		equal	time	repeated One-way ANOVA	F (2.003, 8.011) = 49.30	5	< 0.001
	NES-2min	3	normal		biased	time	Friedman test			< 0.001
3C	control	6	normal	biased		feeding schedule	Kruskal-Wallis test			< 0.001
	NES-5min	5	normal							
	NES-2min	3	normal							
3E	control	6	non-normal			time	Friedman test			< 0.001
	NES-5min	5	normal		biased	time	Friedman test			< 0.001
	NES-2min	3	normal		biased	time	Friedman test			< 0.001
3F	control	6	normal	biased		feeding schedule	Kruskal-Wallis test			< 0.01
	NES-5min	5	normal							
	NES-2min	3	normal							
4B	control	6	non-normal			time	Kruskal-Wallis test			0.751
	NES	7	non-normal			time	Kruskal-Wallis test			0.159
5A-total	control	6	normal	equal		feeding schedule	One-way ANOVA	F (2, 17) = 0.4493	2	0.645
	NES-5min	7	normal							
	NES-2min	7	normal							
5A-ND	control	6	normal	equal		feeding schedule	One-way ANOVA	F (2, 17) = 18.96	2	< 0.001
	NES-5min	7	normal							
	NES-2min	7	normal							
5A-HFD	NES-5min	7	normal	equal		feeding schedule	student's t-test	F (6, 6) = 1.685		< 0.001
	NES-2min	7	normal							

	control	6	normal							
5B	NES-5min	7	normal	equal		feeding schedule	One-way ANOVA	F (2, 17) = 4.678	2	< 0.05
	NES-2min	7	normal							
	control	6	non-normal			time	Friedman test			< 0.001
5C	NES-5min	4	non-normal			time	Friedman test			0.356
	NES-2min	4	non-normal			time	Friedman test			< 0.01
	control	6	normal							
5D	NES-5min	4	normal	biased		feeding schedule	Kruskal-Wallis test			< 0.001
	NES-2min	4	normal							
6B	control	8	normal	biased	equal	time	student's *t*-test	F (7, 7) = 1.398		0.098
	NES	8	normal		biased	time	Mann Whitney test			0.854
6D	control	8	non-normal			time	Mann Whitney test			0.161
	NES	8	non-normal			time	Mann Whitney test			< 0.01
6F	control	6	normal		biased	time	Mann Whitney test			0.937
	NES	7	non-normal			time	Mann Whitney test			0.073
6G	control	6	normal	biased	equal	time	student's *t*-test	F (5, 5) = 5.947		0.301
	NES	7	normal		biased	time	Mann Whitney test			>0.999
6H	control	6	normal	biased	biased	time	Mann Whitney test			0.937
	NES	7	normal		biased	time	Mann Whitney test			0.62
6I	control	6	normal		biased	time	Mann Whitney test			0.393
	NES	7	non-normal			time	Mann Whitney test			0.073
6J	control	6	normal	biased	equal	time	student's *t*-test	F (5, 5) = 5.820		0.317
	NES	7	normal		biased	time	Mann Whitney test			0.209
6K	control	6	normal	biased	biased	time	Mann Whitney test			> 0.999
	NES	7	normal		biased	time	Mann Whitney test			0.805
7B	control	8	non-normal			time	Mann Whitney test			0.959
	NES	8	normal		equal	time	student's *t*-test	F (7, 7) = 2.441		0.561
7D	control	8	non-normal			time	Mann Whitney test			0.721
	NES	8	normal		equal	time	student's *t*-test	F (7, 7) = 1.187		0.109
7F	control	6	non-normal			time	Mann Whitney test			0.065
	NES	7	normal		biased	time	Mann Whitney test			< 0.05

7G	control	6	non-normal			time	Mann Whitney test			0.589
	NES	7	normal		biased	time	Mann Whitney test			0.456
7H	control	6	normal		biased	time	Mann Whitney test			< 0.01
	NES	7	non-normal			time	Mann Whitney test			0.001
7I	control	6	non-normal			time	Mann Whitney test			0.485
	NES	7	normal		biased	time	Mann Whitney test			< 0.05
7J	control	6	non-normal			time	Mann Whitney test			0.394
	NES	7	normal		biased	time	Mann Whitney test			0.259
7K	control	6	normal		biased	time	Mann Whitney test			< 0.05
	NES	7	non-normal			time	Mann Whitney test			< 0.001
S1B-total	control	7	normal			feeding schedule	Kruskal-Wallis test			0.101
	NES-ZT5-HFD	7	normal							
	NES-ZT5-ND	7	non-normal							
	NES-ZT17-HFD	7	normal							
S1B-FF	control	7	normal			feeding schedule	Kruskal-Wallis test			< 0.01
	NES-ZT5-HFD	7	non-normal							
	NES-ZT5-ND	7	normal							
	NES-ZT17-HFD	7	normal							
S1B-RF	NES-ZT5-HFD	7	normal			feeding schedule	Kruskal-Wallis test			< 0.001
	NES-ZT5-ND	7	non-normal							
	NES-ZT17-HFD	7	normal							
S1C	control	7	non-normal			week	Friedman test			0.444
	NES-ZT5-HFD	7	non-normal			week	Friedman test			0.066
	NES-ZT5-ND	7	normal		biased	week	Friedman test			< 0.05
	NES-ZT17-HFD	7	non-normal			week	Friedman test			< 0.05
S1D	control	6	non-normal			time	Friedman test			< 0.001
	NES-ZT5-HFD	7	non-normal			time	Friedman test			< 0.001
	NES-ZT5-ND	7	non-normal			time	Friedman test			< 0.001
	NES-ZT17-HFD	4	non-normal			time	Friedman test			< 0.001
S1E	control	7	normal	equal		feeding schedule	One-way ANOVA	$F(3, 24) = 4.668$	3	< 0.05
	NES-ZT5-HFD	7	normal							
	NES-ZT5-ND	7	normal							

	NES-ZT17-HFD	7	normal							
	control	7	normal							
S2B-total	NES-ZT1	7	normal	equal		feeding schedule	One-way ANOVA	F (2, 18) = 0.8535	2	0.442
	NES-ZT9	7	normal							
	control	7	normal							
S2B-ND	NES-ZT1	7	normal	equal		feeding schedule	One-way ANOVA	F (2, 18) = 2.010	2	0.163
	NES-ZT9	7	normal							
S2B-HFD	NES-ZT1	7	normal	equal		feeding schedule	student's t-test	F (6, 6) = 3.239		0.241
	NES-ZT9	7	normal							
	control	7	non-normal			week	Friedman test			0.241
S2C	NES-ZT1	7	normal		biased	week	Friedman test			< 0.01
	NES-ZT9	7	non-normal			week	Friedman test			< 0.01
	control	7	non-normal			time	Friedman test			< 0.001
S2D	NES-ZT1	7	non-normal			time	Friedman test			< 0.001
	NES-ZT9	7	non-normal			time	Friedman test			< 0.001
	control	7	normal							
S2E	NES-ZT1	7	normal	equal		feeding schedule	One-way ANOVA	F (2, 18) = 0.401	2	0.675
	NES-ZT9	7	normal							
S3A	control	8	non-normal			time	Friedman test			< 0.001
	NES	8	non-normal			time	Friedman test			< 0.001
S3B	control	8	non-normal			time	Friedman test			< 0.001
	NES	8	non-normal			time	Friedman test			< 0.001
S4B	control	5	normal	equal		feeding schedule	student's t-test	F (6, 4) = 2.168		< 0.01
	NES	7	normal							
S4D	control	5	normal	equal		feeding schedule	student's t-test	F (4, 6) = 4.211		0.583
	NES	7	normal							
S6A	control	7	normal	equal		feeding schedule	student's t-test	F (6, 6) = 1.567		0.606
	NES	7	normal							
S6B	control	7	normal			feeding schedule	Mann Whitney test			0.872
	NES	7	non-normal							
S6C	control	8	non-normal			feeding schedule	Kruskal-Wallis test			< 0.05
	NES	8	normal				Kruskal-Wallis test			

S7	control	7	normal	equal		feeding schedule	student's t-test	F (6, 6) = 1.545	0.691
	NES	7	normal						
S8A	control	10	non-normal			time	Wilcoxon signed-rank test		< 0.01
	NES	13	normal			time	paired t-test		< 0.01
S9A-total	control	8	normal	equal		feeding schedule	student's t-test	F (7, 7) = 1.671	0.516
	NES	8	normal						
S9A-ND	control	8	normal	equal		feeding schedule	student's t-test	F (7, 7) = 1.176	< 0.001
	NES	8	normal						
S9B	control	8	non-normal			week	Friedman test		<0.001
	NES	8	normal		biased	week	Friedman test		<0.001
S9C	control	8	normal		biased	time	Friedman test		< 0.001
	NES	8	non-normal			time	Friedman test		< 0.001
S9D	control	8	non-normal			feeding schedule	Mann Whitney test		0.195
	NES	8	normal						
S9E	control	8	non-normal			feeding schedule	Mann Whitney test		< 0.05
	NES	8	normal						
S10C	control	8	non-normal			time	Mann Whitney test		0.328
	NES	8	normal		equal	time	student's *t*-test	F (7, 7) = 1.267	0.33
S10D	control	8	normal		equal	time	student's *t*-test	F (5, 5) = 1.923	0.173
	NES	8	non-normal			time	Mann Whitney test		0.13
S10G	control	6	non-normal			time	Mann Whitney test		0.485
	NES	7	normal		biased	time	Mann Whitney test		0.71
S10H	control	6	normal	biased	biased	time	Mann Whitney test		0.31
	NES	7	normal		equal	time	Mann Whitney test		0.805
S10I	control	6	normal	biased	biased	time	Mann Whitney test		0.132
	NES	7	normal		biased	time	student's *t*-test	F (6, 6) = 0.352	0.748
S10J	control	6	normal	biased	biased	time	Mann Whitney test		0.485
	NES	7	normal		biased	time	Mann Whitney test		0.383
S10K	control	6	normal	biased	biased	time	Mann Whitney test		0.132
	NES	7	normal		biased	time	Mann Whitney test		0.456
S11B	control	8	non-normal			time	Mann Whitney test		0.065
	NES	8	non-normal			time	Mann Whitney test		0.382

S11D	control	8	non-normal			time	Mann Whitney test		0.798
	NES	8	non-normal			time	Mann Whitney test		0.959
S11F	control	6	normal	biased	biased	time	Mann Whitney test		0.18
	NES	7	normal		biased	time	Mann Whitney test		< 0.05
S11G	control	6	non-normal			time	Mann Whitney test		0.699
	NES	7	normal		equal	time	student's *t*-test	F (6, 6) = 5.277	0.698
S11H	control	6	normal	biased	biased	time	Mann Whitney test		0.394
	NES	7	normal		equal	time	student's *t*-test	F (6, 6) = 4.348	< 0.05
S11I	control	6	normal	biased	biased	time	Mann Whitney test		< 0.01
	NES	7	normal		biased	time	Mann Whitney test		0.383
S11J	control	6	non-normal			time	Mann Whitney test		0.485
	NES	7	normal		biased	time	student's *t*-test	F (6, 6) = 3.828	0.864
S11K	control	6	normal	biased	biased	time	Mann Whitney test		0.0645
	NES	7	normal			time	student's *t*-test	F (6, 6) = 4.288	< 0.05
